# Measuring Emotion Perception Ability Using AI-Generated Stimuli: Development and Validation of the PAGE Test

**DOI:** 10.3390/jintelligence13090116

**Published:** 2025-09-10

**Authors:** Ben Weidmann, Yixian Xu

**Affiliations:** 1 Social Research Institute, University College London, Gower St., London WC1E 6BT, UK; 2 Harvard Kennedy School, Harvard University, Cambridge, MA 02138, USA; yixian_xu@hks.harvard.edu

**Keywords:** emotion perception, teamwork, management, measurement, generative AI

## Abstract

We present a new measure of emotion perception called PAGE (Perceiving AI Generated Emotions). The test includes 20 emotions, expressed by ethnically diverse faces, spanning a wide range of ages. We created stimuli with generative AI, illustrating a method to build customizable assessments of emotional intelligence at relatively low cost. Study 1 describes the validation of the image set and test construction. Study 2 reports the psychometric properties of the test, including convergent validity and relatively strong reliability. Study 3 explores predictive validity using a lab experiment in which we causally identify the contributions managers make to teams. PAGE scores predict managers’ causal contributions to group success, a finding which is robust to controlling for personality and demographic characteristics. We discuss the potential of generative AI to automate development of non-cognitive skill assessments.

## 1. Introduction

Emotion perception ability refers to individual differences in the ability to accurately detect and label others’ emotions from nonverbal channels such as the face, voice, and body (Schlegel et al. 2014). In the ability model of emotional intelligence, perceiving emotions represents a foundational skill that enables downstream abilities like understanding and regulating emotions (Joseph and Newman 2010; Salovey and Mayer 1990). Emotion perception ability also constitutes the emotion-focused subdomain of interpersonal accuracy, the broader capacity to make accurate judgments about others’ internal states and traits, including attitudes, intentions, and personality (Hall and Bernieri 2001). Accurately recognizing others’ emotions allows individuals to disambiguate social situations, and respond appropriately to others’ actions, facilitating effective social interaction and coordination (Keltner and Haidt 1999; van Kleef 2016).

The ability to recognize the emotional states of others also matters for workplace success. Empirical research has shown that emotion perception predicts income, job performance (Côté 2014; Elfenbein 2023), leadership emergence (Côté et al. 2010), teamwork effectiveness (Farh et al. 2012; Weidmann and Deming 2021; Riedl et al. 2021) and successful negotiation outcomes (Elfenbein et al. 2007; Schlegel et al. 2018).

Researchers began developing skill-based tests to measure emotion perception in the 1970s (Martin et al. 1996). These tests generally ask participants to assess emotional expressions that are portrayed by actors in videos (e.g., Geneva emotion recognition test, Schlegel and Scherer 2016), images (e.g., Reading the Mind in the Eyes test, Baron-Cohen et al. 2001) or audio recordings (e.g., DANVA-2, Nowicki and Duke 1994). The assessments have been used in a wide range of disciplines including psychology (Elfenbein et al. 2007), economics (Caplin et al. 2023) and medicine (Kohler et al. 2003).

However, existing measures face four challenges that may limit their usefulness (summarized in Table 1). First, most tests use ethnically homogenous stimuli and often only include Caucasian faces (Bell et al. 1997; Baron-Cohen et al. 2001; Mayer et al. 2003; Scherer and Ellgring 2007b; Bänziger et al. 2011; Schlegel and Scherer 2016; Scherer and Scherer 2011). This can result in biased tests, as participants recognize emotions more quickly and accurately when the person expressing the emotions shares their cultural and ethnic identity (Elfenbein and Ambady 2003; Young et al. 2012).^1^ Ethnically homogenous test stimuli are especially problematic when assessments are used in a diverse workforce. Second, many assessments lack emotional range and encompass only a handful of basic emotions—often including only one positive emotion (Bell et al. 1997; Mayer et al. 2003; Scherer and Scherer 2011; Nowicki and Duke 1994; Matsumoto et al. 2000; Kohler et al. 2003). This can lead to ceiling effects and also may limit the external validity of tests, as real-world contexts involve a wide range of complex emotions, such as ‘confusion’ (van Kleef 2016; Phillips and Slessor 2011; Rozin and Cohen 2003). Third, many existing tests have practical limitations that make them difficult for researchers to use, including the length (Bell et al. 1997; Bänziger et al. 2011; Schlegel and Scherer 2016; Scherer and Scherer 2011; Bänziger et al. 2009) and cost of tests (Mayer et al. 2003; Nowicki and Duke 1994; Matsumoto et al. 2000), along with the lack of freedom to use assessments on whatever platform researchers find convenient (Schlegel and Scherer 2016). Finally, many existing emotion perception assessments have not yet demonstrated predictive validity in teamwork or workplace settings, limiting their empirical usefulness for management and teamwork research.

To address these limitations, our paper develops a practical test that includes a wide range of emotions expressed by racially diverse faces, spanning ages 20 to 60. We then examine the predictive validity of the test in the context of managing a team. Existing evidence has shown that generative AI can produce test items for domains such as algebra (Bhandari et al. 2024) and medical exams (Kaya et al. 2025; Artsi et al. 2024), achieving psychometric properties comparable to human-written items. In our study, we provide initial evidence that generative AI can also create facial stimuli, and assist researchers in constructing customizable assessments of emotion perception at relatively low cost. Models such as OpenAI’s DALL-E and Google’s Imagen are capable of generating photorealistic images using simple text prompts (Ramesh et al. 2022; Saharia et al. 2022). We use DALL-E to develop our diverse emotion recognition assessment called ‘Perceiving AI Generated Emotions (PAGE)’.

Ultimately, the PAGE test was designed with the goal of assisting researchers who study the role played by emotion perception in real-world social interactions, especially those requiring teamwork.

The rest of the paper proceeds as follows. Study 1 describes the construction of the test. Studies 2a and 2b assess its psychometric properties and examine convergent validity by reporting on the correlation between ‘Perceiving AI Generated Emotions (PAGE)’ and ‘Reading the Mind in the Eyes Test (RMET)’, a widely-used measure of emotion perception and theory of mind (Baron-Cohen et al. 2001). Study 3 explores the predictive validity of the PAGE test. Using repeated random assignment of managers to groups, we examine the extent to which PAGE predicts the causal contribution that managers make to team success.

## 2. Study 1: Test Construction and Image Validation

This section describes the process of constructing the PAGE assessment, including our approach to generating and validating images. This initial validation process involved a study of *N* = 500 participants (Study 1).

### 2.1. Emotion Selection

Many emotion perception tasks are limited to the “basic six emotions”—anger, disgust, fear, happiness, sadness, and surprise (Ekman et al. 1969). Recent research suggests that people can reliably recognize up to 28 emotions from facial–bodily expressions (Cowen and Keltner 2020). We only include emotions that are not dependent on bodily expression and contextual clues. The 25 emotions are six basic emotions (Happiness, Anger, Fear, Anger, Disgust, Surprise), and 19 complex emotions: Disappointment (Cordaro et al. 2016), Amusement (Keltner 1995), Anxiety (Perkins et al. 2012), Awe (Shiota et al. 2017), Boredom (Scherer and Ellgring 2007a, 2007b; Cordaro et al. 2020), Concentration, Confusion, Contemplation (Rozin and Cohen 2003), Contempt (Matsumoto and Ekman 2004), Contentment (Cordaro et al. 2016), Desire, Doubt, Embarrassment (Keltner 1995), Interest (Reeve 1993), Pain (Prkachin 1992), Pride (Tracy and Robins 2004), Relief, Shame (Tracy and Matsumoto 2008), Sympathy (Goetz et al. 2010).

### 2.2. Generating Emotional Faces

Face stimuli were generated using DALL-E 2, a diffusion-based model that allows users to generate photorealistic images from text prompts (Ramesh et al. 2022; Marcus et al. 2022). We selected DALL-E 2, as diffusion-based models have been shown to generate higher-quality facial images compared to GANs in previous studies (Stypułkowski et al. 2023).

The prompts we gave DALL-E 2 used three methods, derived from emotion elicitation strategies researchers use when creating emotional stimuli using human actors. The first method is simply to instruct expressers to express a particular emotion (as used, for example, in ‘Karolinska Directed Emotional Faces (KDEF)’ and NimStim (Lundqvist et al. 1998; Tottenham et al. 2009), e.g., ‘a 22 year old Caucasian woman feeling very angry.’ The second method relies on the Directed Facial Action Task (Ekman 2007), in which expressers are instructed to employ specific facial actions based on the emotion prototypes identified by Ekman and colleagues (Ekman 1992). For example, to express the emotion pride we prompted DALL-E 2 by saying ‘a 30 year old Asian man showing pride. His head is held high, jaw thrust out, he has a small smile, lip pressed.’ Finally, we borrow a technique from studies of cultural variation in emotional expressions and use a short story to elicit emotions in expressers (Cordaro et al. 2018). For example, to generate an image of surprise, the prompt includes the following text ‘a 47 year old Indian woman showing a surprised face when hearing a loud sound she didn’t expect.’

We experimented with a combination of these three methods to elicit emotions in AI images, operationalized as prompts in three formats: ‘emotion word’, ‘emotion word, and facial actions’ and ‘emotion word, and one-sentence emotion story.’ To generate human-like images, each prompt begins with ‘Generate a photorealistic image of…’. We also added ‘detailed skin texture’, and ‘proportional eyes’ into the prompt, which are found to be among the key factors in making an AI face look more realistic (Miller et al. 2023). An example prompt that uses all three of the techniques listed above is as follows: ‘A realistic photo of a 20 year old Indian woman caught embarrassed and blushing in a social gaffe. Her whole face and head are in the middle. Plain grey background (leave some blank space around). She is wearing a white t-shirt. No body language, head oriented at the front, and staring at the camera.’ A full list of prompts is provided for each emotion in Supporting Information, Table S3.

**Figure 1:**
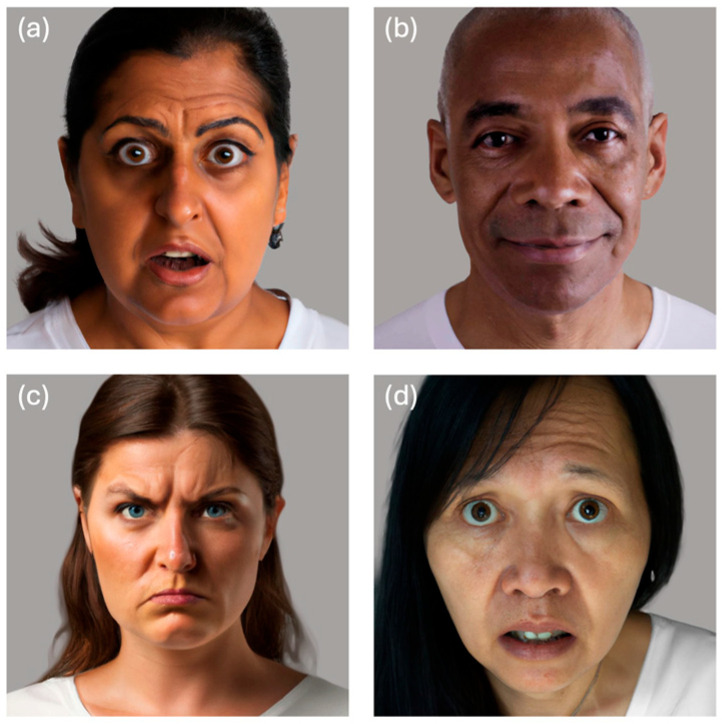
Example Stimuli from the PAGE test. In this case, the emotions being represented by the four stimuli are (**a**) surprise, (**b**) contentment, (**c**) anger, (**d**) fear.

We generated 150 realistic faces for the initial stim set. These faces represent 25 emotions, six ethnicities, and ages ranging from 20 to 60. We used the same ethnicity categories as the Chicago Face Database: Asian, Black, Caucasian, Indian, Latino, Multi-racial (Ma et al. 2015). We then used Adobe Photoshop to standardize the stimuli to have consistent grey background. Images were resized so that each target’s face and head are in the middle. See Figure 1 for sample stimuli.

### 2.3. Validation and Selection of Stimuli

We recruited 500 participants on Prolific to rate the stimuli in emotion categories. Each participant rated between 30 and 35 images. Participants were asked to select one emotion that best described the face, from a list of 25 emotions. Each image was rated by at least 100 participants. Our sample was ethnically diverse and displayed gender balance (female 49%, mean age 34 years, White 57.8%, see Appendix A, Table A1 for full demographic statistics). Each participant received a compensation of 2 USD for completion of the task.

For each candidate image we computed the proportion of participants selecting the prompted (target) emotion, out of 25 possible options. We retained an image when the target emotion was the most frequently chosen option. For example, for stimulus Amusement_35_Caucasian_Male, 77.3% out of 110 raters selected ‘Amusement,’ exceeding any other options. A confusion matrix reporting the percentage of participants who selected each emotion for every image, is provided in the Supporting Information (Table S1). Using the 40 retained images, we constructed preliminary versions of the PAGE test and conducted iterative pilot studies to evaluate item- and test-level psychometrics (e.g., difficulty, item–total correlations, factor loadings). Poorly performing items were revised or removed, resulting in a final stimulus set of 35 images. Figure S1 of Supporting Information summarizes the development pipeline. Details of the item selection criteria are described in the next section.

### 2.4. Test Construction

We generated a multiple-choice question for each image by selecting five distractors. These distractors were drawn from two main sources: first, the emotion labels chosen by participants during the stimuli validation task; second, other plausibly relevant emotions from the 25 emotions included in the test (Cowen and Keltner 2020). Additionally, emotions which are frequent parts of social interactions such as confusion, doubt, and interest (Benitez-Quiroz et al. 2016; Rozin and Cohen 2003) are overrepresented in the distractors.

The preliminary version of the PAGE test has 40 items; we then conducted iterative pilot studies to evaluate item- and test-level psychometrics. Several considerations guided the refinement of the test and the selection of the final item set: (1) the overall test difficulty should avoid floor and ceiling effects, ensuring an appropriate level of challenge; (2) each item should demonstrate a positive item–total correlation with the overall test score; and (3) the final item set should represent racially diverse faces, maintain gender balance, and include a broad range of basic and complex emotions. Ultimately, we retained 35 items for the final version of the PAGE test (see Appendix A, Table A2 for the demographic characteristics of the images). Detailed item characteristics, including item–total correlations and factor loadings, are provided in Table S4 of the Supporting Information, item difficulty is reported in Table S3. A full list of face stimuli, target emotions, and distractors for each item is provided in Supporting Information, Table S2.

**Figure 2:**
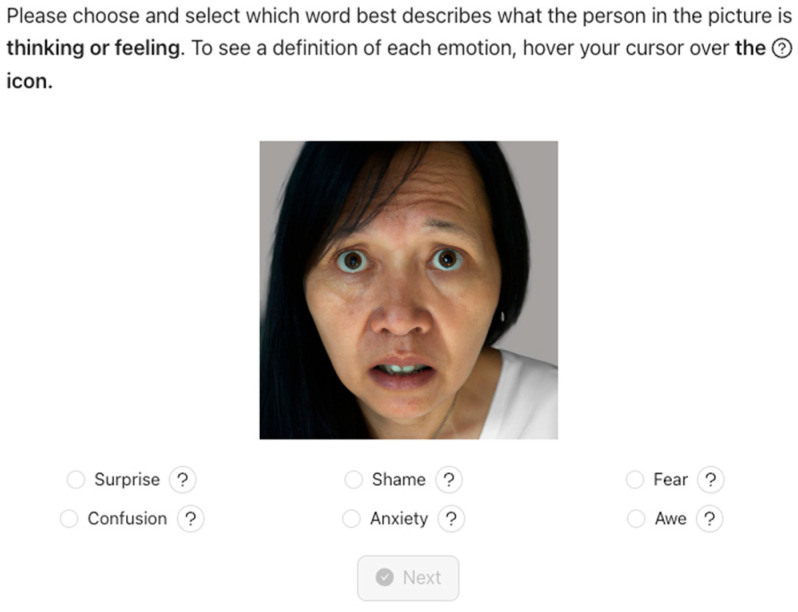
Sample item from the PAGE test. For each item, participants select one answer from six options. Definitions of emotions are provided to participants if they click on the question marks. The target emotion for this item is fear.

The resulting set of 35 test questions were sequenced such that consecutive items did not feature the same emotion. The placement of both the correct emotion and the distractors were randomized. Correct answers are scored as 1, incorrect as 0. All materials are freely available. We also made both the short and full versions of the PAGE task publicly accessible via our lab website. Detailed descriptions of the construction of the short PAGE, and both instruments are provided in the Supporting Information. See Figure 2 for an example item of the PAGE test.

## 3. Study 2a: Measurement Properties of PAGE

### 3.1. Participants and Procedure

We recruited 1010 participants from Prolific. Participants provided written informed consent to take part in the study with data collected solely for research purposes. All participants were located in the United States and were ethnically diverse (female 50%, mean age 36.7 years, White 44.5%, see Appendix A, Table A1 for demographic details). We oversampled non-Caucasian participants so that we could better assess performance on the PAGE of people from different ethnic backgrounds. We also focused on respondents who were full-time workers (91%) aged 25–55 to validate the PAGE among a sample that would be of use to labor economists and organizational psychologists. We administered the PAGE test on Qualtrics (full instructions in Supporting Information) and each participant received a compensation of 2.50 USD. To motivate participants to maintain attention throughout the task, we also awarded the top quintile of performers a 5 USD bonus. The median participant spent 8 min on the test.

### 3.2. Results

**Figure 3:**
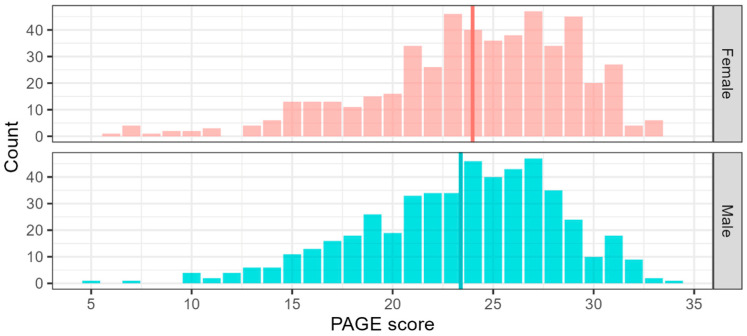
Distribution of PAGE scores by gender (*N* = 505 males and 505 females). Mean score for male is 23.4 (*SD* = 4.9), Mean score for female is 24.0 (*SD* = 5.1), mean scores indicated by the vertical lines. The mean difference between males and females is not statistically significant.

**Figure 4:**
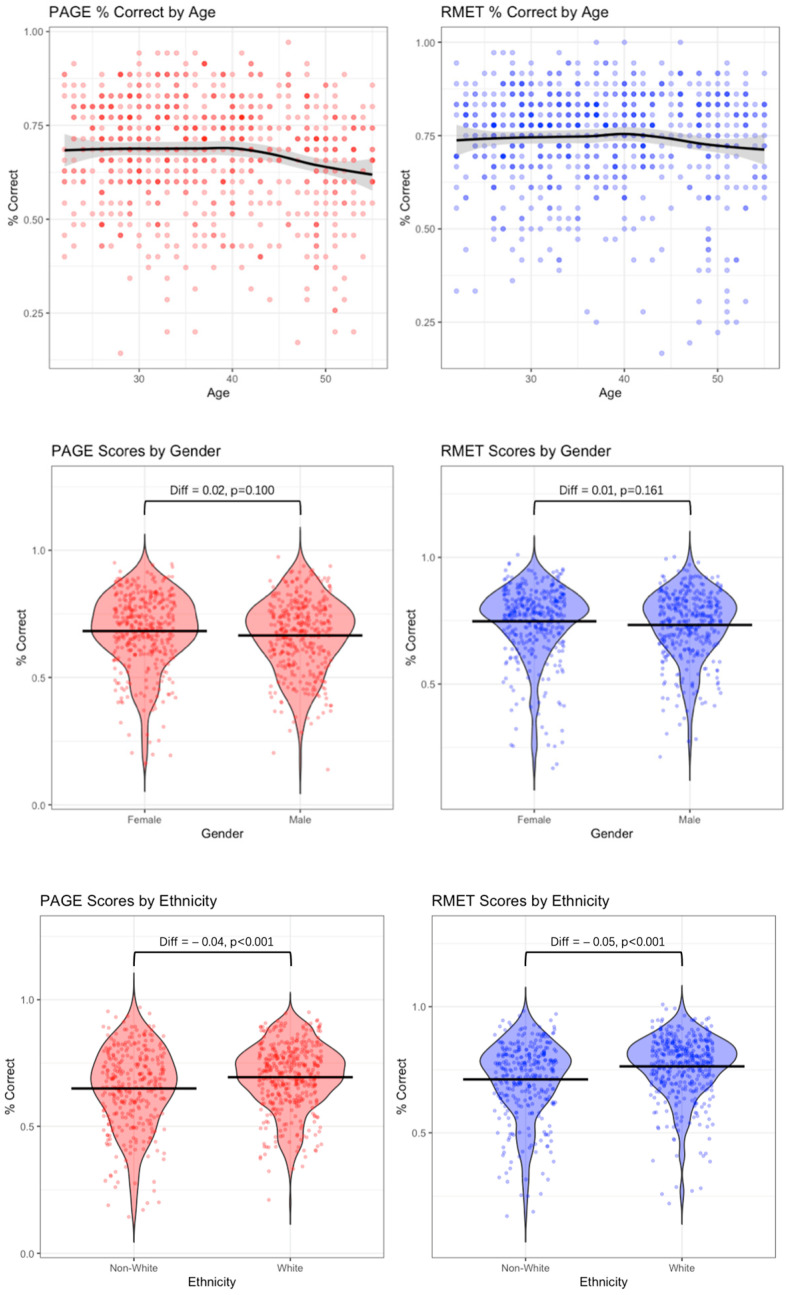
Patterns of performance for PAGE/RMET by gender/age/ethnicity (*N* = 741). 50% of the participants are female, and 50.5% are White. Both tests show similar performance patterns across gender, age and ethnicity. Each dot on the graphs represents one observation.

The mean score for PAGE is 23.7 (*SD* = 5.0). There was no evidence of ceiling or floor effects, and no statistically significant difference in task performance between men and women, as shown in Figure 3. There was a negative correlation between PAGE scores and age (r = −0.14, *p* < 0.001), as shown in Figure 4. These findings are consistent with previous work showing lower accuracy at emotional recognition in older adults (Mill et al. 2009; Ruffman et al. 2008).

**Item difficulty.** The mean item difficulty—measured in terms of the proportion of people submitting correct answers—was 0.68 (*SD* = 0.12). Individual item difficulties ranged from 0.44 to 0.89; this range falls within recommended parameters for psychological assessment measures (Thorndike and Thorndike-Christ 2010). Table 2 presents the distribution of item difficulties. A full table of item difficulty across items and populations is available in Supporting Information, Table S2.

**Internal consistency.** Cronbach’s alpha for the PAGE test was 0.73,^2^ compared to an average reliability of α = 0.60 reported for other emotion recognition ability tests (Boone and Schlegel 2016). This is notable given the brevity of the test—8 min on average—and the wide range of emotions included in the test, both of which tend to reduce internal consistency.

**Figure A1:**
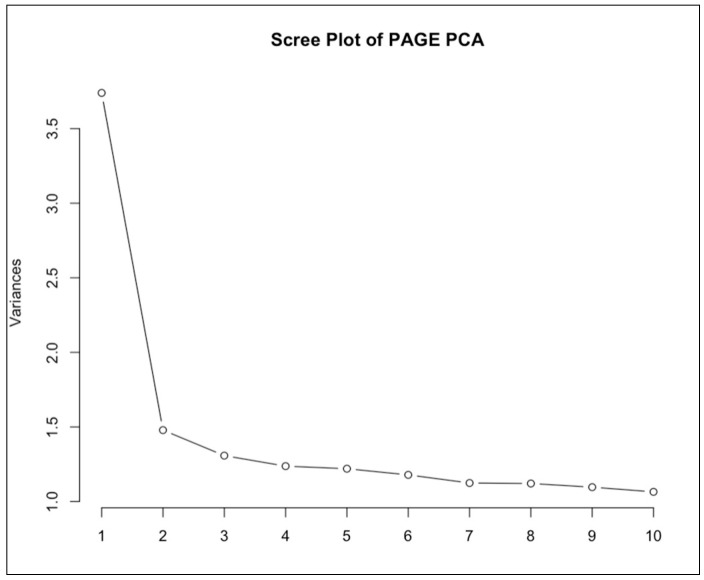
Study 2a: Scree plot of PAGE. The plot displays the eigenvalues for the components extracted with principal component analysis (PCA). The sharp drop between the first and second components indicates a dominant one-factor structure.

**Factor structure.** Existing evidence has shown that emotion perception ability is a unidimensional construct (Connolly et al. 2020; Schlegel et al. 2014; Schlegel and Scherer 2016). An item-level principal component analysis (PCA) scree plot suggests that the PAGE test has a one-factor structure (Appendix B, Figure A1). Additionally, we conducted Velicer’s MAP test, which indicated a 1-factor solution (minimum average partial correlation = 0.01); very simple structure (VSS) analysis also supported a 1-factor solution for complexity 1 (maximum = 0.52 at 1 factor). To further assess the unidimensionality of the PAGE instrument, we conducted a confirmatory factor analysis (CFA) using 12 item parcels, each comprising 2–3 items grouped by item difficulty.^3^ This approach reduces measurement noise and allows for more stable estimation of the factor structure (Little et al. 2002). The one-factor CFA model, estimated with the WLSMV estimator, showed excellent fit: χ^2^(54) = 78.78, *p* = 0.016, robust CFI = 0.975, robust TLI = 0.969, robust RMSEA = 0.021. These indices indicate a strong fit to a unidimensional structure.^4^

## 4. Study 2b: Convergent Validity of PAGE

### 4.1. Reading the Mind in the Eyes Test (RMET)

To demonstrate convergent validity, we compare results on the PAGE test to the Reading the Mind in the Eyes Test (RMET). The RMET is a 36-item multiple choice test measuring emotion perception by presenting cropped images of faces that only include the eye region (Baron-Cohen et al. 2001).

We also examine associations with age, gender, and ethnicity because prior research documents systematic demographic patterns in emotion perception ability. Meta-analyses show that women exhibit a small but reliable advantage in recognizing emotional expressions (Thompson and Voyer 2014), whereas older adults perform worse than younger adults (Ruffman et al. 2008; Mill et al. 2009). In addition, accuracy is higher when perceivers and expressers share racial/ethnic or cultural group membership (an in-group advantage) (Elfenbein and Ambady 2002). Because widely used instruments such as the RMET have historically employed Caucasian-only stimuli, such tests can inadvertently favor White participants; using racially diverse face stimuli is expected to mitigate these biases (Conley et al. 2018). We therefore compare demographic correlations for both PAGE and RMET to assess convergence and potential attenuation of ethnicity-linked advantages (Figure 4).

### 4.2. Participants and Procedure

We analyze a sub-sample of 741 participants from Study 2a, who completed both PAGE and RMET, administered on Qualtrics. Participant demographics are presented in Appendix A, Table A1. Both tasks included one practice question to familiarize participants with the task format. To limit the effect of differences in vocabulary, we provided a list of emotion definitions for reference. When participants put their cursor above the emotion word, they were provided with a definition. To reduce the impact of order effects, we had 249 participants complete the RMET first (then the PAGE) and 492 people complete the tests in the reverse order. Question order was also randomized for both tests. On average, participants completed the PAGE in 8 min and the RMET in 10 min.

### 4.3. Results

We find that the PAGE is highly correlated with RMET (raw correlation = 0.66, disattenuated correlation = 0.88, *p* < 0.001), providing evidence of convergent validity. We also explored the patterns of performance between PAGE and RMET by age, gender, ethnicity, shown in Figure 4. The PAGE test shows the same performance patterns as RMET. First, women perform slightly better than men, though the difference is not statistically significant for both tests. Second, both tests show stable performance from age 18 to 40, then a slight decline up to age 60, where our sample ends. Last, mean performance of Caucasian participants is slightly better on both tests, but the advantage is attenuated in the performance of PAGE test.

## 5. Study 3: Predictive Validity of the PAGE Assessment

### 5.1. Participants

We recruited an ethnically diverse sample of graduate and undergraduate students at the University of Essex in the UK. Participants provided written informed consent to take part in the study. The median participant was 25 years old and had 2 years’ work experience (Appendix A, Table A1). Participants were paid 29 GBP for completing the study, with a performance bonus that ranged from 0 to 12 GBP. The average payment was 35 GBP.

### 5.2. Experiment Procedures

**Figure 5:**
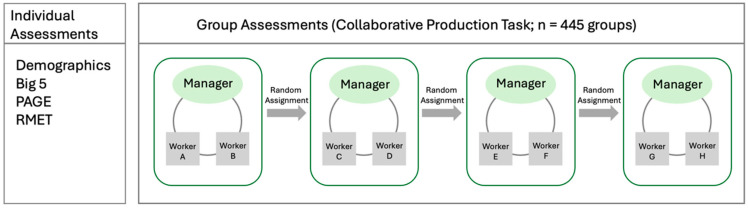
Experiment overview. Participants were randomly assigned to the role of ‘manager’ or ‘worker’. Each group completed a novel collaborative problem-solving task in which managers assigned tasks, monitored group progress, and motivated teammates. After each task, managers were randomly reassigned to new groups, managing a total of four different groups. The repeated random assignment of managers to teams enabled the identification of each manager’s contribution to group performance.

To assess the predictive validity of PAGE we fielded the test in a lab experiment that used a novel design to identify the causal contribution that individual managers make to group performance. The experimental design—summarized in Figure 5—randomly assigns managers to four different teams of workers. Our design makes use of the repeated random assignment of managers to teams to identify the average impact each manager has on group performance (Weidmann and Deming 2021). Each team consists of a manager and two workers. Teams work face-to-face on a collaborative task that aims to emulate some of the core demands of real-world hierarchical teams by requiring managers to co-ordinate, monitor and motivate workers. The task is described in detail in (Weidmann et al. 2024). Briefly, teams are asked to simultaneously make progress on three different ‘modules’ (with one module each for numerical, spatial and analytical questions). The group’s final score is based on a ‘weakest-link’ scoring rule (Hirshleifer 1983). Specifically, the final score is the score of the module with the fewest points. With this setup, a central responsibility of the managers is to make decisions about which module each member of the team—including themselves—should work on. Decisions about task allocations are fully dynamic and managers can change them at any time. The group task takes around 15 min in total and includes dedicated time for managers to introduce themselves and motivate their team. Talking is allowed throughout the task. After each group finished the task, managers are randomly assigned to another team. Over the course of the experiment each manager is randomly assigned to four groups.

To succeed in the task, managers have to assign their teammates tasks that match their skills, monitor their performance and maintain high levels of effort and engagement (Weidmann and Deming 2021). We hypothesize that managers with strong emotional perception may be better placed to meet these managerial demands (Rodrigues and Matos 2024; Castro et al. 2022; Acheampong et al. 2023). For example, a manager skilled at detecting boredom may be faster at perceiving low morale and better able to respond by providing encouragement or switching the task a teammate is working on. Similarly, a manager who can perceive that a teammate is confused is in a better position to offer support and avoid their teammate submitting incorrect solutions on behalf of the team. A total of 115 managers in the experiment completed the PAGE instrument, which allowed us to compare PAGE scores with causally identified manager contributions. These participants also completed the Reading the Mind in the Eyes Test (RMET), providing a benchmark for comparison.

### 5.3. Results

First, we note that the correlation between PAGE and RMET in this sample is high (raw correlation = 0.50, disattenuated correlation = 0.66, *p* < 0.001) although not as high as the Prolific sample. We also note that the mean score on PAGE in the study was 21.5 (*SD* = 4.7), slightly lower than the mean for the Prolific sample (23.7 out of 35).

We find that the PAGE score of each manager is positively associated with group performance. The correlation between manager PAGE scores and group performance = 0.183 (*p* < 0.001, *N* = 408 groups).^5^ These results are consistent with findings from non-hierarchical teamwork settings where team members’ emotion perception ability positively predicts team success (Weidmann and Deming 2021; Elfenbein et al. 2007).

Next, we move from the group level to the level of individual managers. We identify and estimate the average causal impact each manager has by exploiting the fact managers are randomly assigned to multiple teams (Weidmann and Deming 2021).^6^ We find that PAGE scores positively predict the average causal contribution that managers have on their groups (correlation = 0.290, *p* = 0.002, df = 113).

Table 3 contextualizes PAGE’s association with managerial causal contributions by comparing its predictive validity to RMET. Overall, we find that the association between PAGE scores and manager contributions is greater than that for RMET, and that the association is robust to controls for differences in Big 5 personality measures (Gosling et al. 2003) and demographic factors (age, gender, ethnicity and education). Column 1 (Table 3) presents the raw association between manager causal contribution and PAGE scores, which are standardized to have mean = 0 and *SD* = 1. We find that a 1sd increase in PAGE scores is associated with an increase in manager contributions of 0.290sd. Columns 2 and 3 add controls for Big 5 personality and demographics which reduces the coefficients, but they remain significant: after controlling for age, ethnicity, gender, education program and Big 5 measures, a 1sd change in PAGE is associated with a 0.230sd increase in managerial contributions (*p* = 0.031). Columns 4 to 6 repeat this process, focusing on RMET as a predictor. The relationship between RMET and manager causal contributions are weaker, and not statistically significant. Column 7 is a full specification in which we include all variables, illustrating the robust association between PAGE scores and the impact that managers have on their teams.

Why might PAGE be more predictive of managerial performance than RMET? One possibility is that the emotions in the PAGE assessment were chosen from a list of emotions that can be clearly expressed by faces (Cowen and Keltner 2020). In contrast, some of the target emotions in RMET may be better characterized as dispositions (e.g., ‘cautious’) that are difficult to express and perceive in faces. We also explored the possibility that the predictive performance of PAGE was enhanced by the fact that Study 3’s participant sample is ethnically diverse and may have benefited from PAGE’s more diverse set of stimuli. To examine this empirically we split teams into two sets: ethnically homogenous (all group members self-identify with the same ethnicity) and ethnically diverse (at least two group members self-identify with different ethnicities). For each set we separately calculate the association between team performance and the manager’s score on RMET|PAGE. Among diverse groups, the PAGE test significantly predicted performance ($\hat{\rho }$ = 0.216, *p* < 0.001, *N* = 342). This was not the case for homogenous groups ($\hat{\rho }=\;-$0.002, *p* = 0.985, *N* = 66). The correlation between RMET and group performance was not statistically significant for either homogeneous or diverse groups.

Finally, we return to the question of how a manager’s emotion perception might improve team performance. As noted above, one of the roles of the manager is to motivate their teammates. Motivation matters in Study 3’s collaborative task for two reasons. First, participants are given a large number of cognitively demanding puzzles that require effort to solve. Second, ‘workers’ in the experiment do not receive financial incentives based on performance, so they are prone to lose interest over the course of the task. As noted in Weidmann et al. (2024), the task involves three periods of intensive problem solving, divided by two dedicated breaks in which managers can take stock and motivate their team.

To explore the role that motivation plays in the task, we separately calculate the causal contribution that managers make in each of the three problem-solving periods (i.e., start; middle; end). Weidmann et al. (2024) find that the last period matters most in terms of managerial contribution. We extend this finding by documenting that the PAGE test is most strongly predictive of performance in the final period of the task, as noted in Table 4. Column 1 in the table regresses each manager’s average causal contribution during the first period of the task against their PAGE score. Column 2 repeats the exercise, focusing on the average causal contribution managers make during the middle period of the task. Column 3 presents results for the final period. A one standard-deviation increase in the PAGE test is associated with a 0.235 standard-deviation increase in managerial contributions in the final period (*N* = 87, *p* = 0.022). The association between PAGE and managerial contributions in the first and second periods are not statistically significant.

## 6. Discussion, Limitations and Conclusions

This paper develops and validates a measure of emotion perception using a demographically diverse set of 35 faces, expressing 20 emotions. The PAGE test materials are open source. Study 1 shows that generative AI is capable of producing standardized, realistic faces that express both basic and complex emotions. We note that this approach has clear limitations in that it presents static facial expressions and does not incorporate dynamic or multimodal cues (such as vocal expressions) which are important to emotion perception in real-world interactions (Ambadar et al. 2005; Schlegel et al. 2014).^7^ This may limit the test’s predictive validity. Studies 2a and 2b demonstrate the psychometric properties of the test including unidimensionality, internal validity and convergent validity. While these results are generally strong relative to other measures of emotional perception, we note that convergent validity of PAGE relies on one existing test (RMET) which itself has received methodological criticism (Higgins et al. 2023).^8^ Finally, Study 3 provides initial evidence of predictive validity, especially for researchers interested in leadership and management, by showing that PAGE scores are associated with the causal contribution that managers make to group performance in a controlled lab study. A limitation of this study is that it does not control for fluid intelligence, which may be an important predictor of managerial contributions (Dasborough et al. 2022). Overall, these results suggest that PAGE may be useful for researchers looking for a short, skill-based test of emotion perception that is suited for studies of teamwork and management among demographically diverse populations.

The PAGE assessment also illustrates the possibility that generative AI can help create customized measures of emotional intelligence by substantially reducing the cost of test creation and automating the test development process. There are three ways in which tests may be usefully customized. First, researchers may find it helpful to be able to vary the demographic profile of the stimuli. For example, researchers working with a sample of elderly adults may want a test in which stimuli have older faces than are found in existing assessments. Second, generative AI could be used to vary the emotional intensity of stimuli, providing researchers with a way of controlling the difficulty of assessments.^9^ Last, it may be beneficial to have tests that oversample specific complex emotions—many of which are absent from most measures. For example, an organization hiring a team leader may screen for the ability to recognize confusion, as this potentially enables quick clarification. In contexts where teamwork is important, perceiving anxiety may signal a colleague’s need for support. Of course, it is an empirical question whether such customized measures of emotional recognition are more predictive of positive outcomes in real-world contexts, but with the advent of generative AI this research agenda is much more practically achievable.

Improvements in AI technology may further reduce the costs of creating tests of emotion perception. We manually created multiple-choice questions for the PAGE test. However, generative AI is now capable of creating multiple-choice questions across difficulty levels (Aryadoust et al. 2024). More significantly, recent research suggests the possibility that large language models (LLMs) may be able to produce similar results to human participants in social science research (Horton 2023). If this is true of emotional perceptivity, test developers would be able to combine human responses with a low-cost sample of LLM respondents to assess and refine the psychometric properties of new tests. Overall, while existing AI technology reduced the practical barriers we faced in creating PAGE, it seems likely that these barriers will be progressively lowered.

In closing, we believe that the PAGE test measures a general construct that is an important determinant of success in a wide range of social activities, from negotiation and hiring, to networking and working in a team. The test has strong measurement properties, is appropriate for diverse populations, and is open access. We hope that others will build on the approach of using generative AI to create and validate customized tests that allow for a better understanding of the role emotion plays in facilitating interaction in the workplace and beyond.

## Figures and Tables

**Table 1:** Existing measures of emotional perception and their potential limitations.

Test	Emotional Range	Ethnic Diversity	Practical Challenges	Number of Items
DANVA-2 (Nowicki and Duke 1994)	4 emotions	Caucasian, Black	Not freely available	48
BLERT (Bell et al. 1997)	7 emotions	Caucasian	15–20 min	21
JACBART (Matsumoto et al. 2000)	7 emotions	Asian, Caucasian	Not freely available	56
RMET (Baron-Cohen et al. 2001)	26 mental states	Caucasian	None	36
PERT-96 (Kohler et al. 2003)	5 emotions	Diverse	None	96
MSCEIT Perception Tests (Mayer et al. 2003)	5 emotions	Caucasian	Not freely available	50
MERT (Bänziger et al. 2009)	10 emotions	Caucasian	45–60 min	120
MiniPONS (Bänziger et al. 2011)	2 affective situations	Caucasian	15–20 min	64
ERI (Scherer and Scherer 2011)	5 emotions	Caucasian	15–20 min	60
GERT-S (Schlegel and Scherer 2016)	14 emotions	Caucasian	15–20 min; No customization	42
MET (LaPalme et al. 2023)	17 emotions	Diverse	15–20 min	64
MRMET (Kim et al. 2024)	18 mental states	Diverse	None	37 or 10

Notes: DANVA: Diagnostic Assessment of Non-Verbal Abilities; BLERT: Bell Lysaker Emotion Recognition Task; JACBART: Japanese and Caucasian Brief Affect Recognition Test; RMET: The Reading the Mind in the Eyes Test; PERT-96: Penn Emotion Recognition Task; MSCEIT: Mayer–Salovey–Caruso Emotional Intelligence Test; MERT: Multimodal Emotion Recognition Test; MiniPONS: Profile of Nonverbal Sensitivity (short version); ERI: Emotion Recognition Index; GERT-S: Geneva Emotion Recognition Test (short version); MET: Meso-Expression Test; MRMET: Multiracial Reading the Mind in the Eyes Test.

**Table 2:** Distribution of item difficulty. ‘Difficulty range’ indicates the range of proportion of correct responses (‘*p*’).

Difficulty Range	Number of Items
0.30 ≤ *p* < 0.50	3 (8.6%)
0.50 ≤ *p* < 0.70	18 (51.4%)
0.70 ≤ *p* < 0.90	14 (40%)

**Table 3:** Associations between manager performance and emotion perception measured by PAGE vs. RMET.

	Average Causal Contributions of Managers						
	(1)	(2)	(3)	(4)	(5)	(6)	(7)
PAGE	0.303 **	0.241 *	0.230 *				0.273 *
	(0.095)	(0.096)	(0.105)				(0.113)
RMET				0.146	0.067	−0.025	−0.125
				(0.092)	(0.093)	(0.111)	(0.116)
Big5		X	X		X	X	X
Demographics			X			X	X
Observations	109	109	109	109	109	109	109
*R* ^2^	0.088	0.171	0.223	0.023	0.124	0.184	0.233
Adjusted *R*^2^	0.079	0.122	0.117	0.014	0.073	0.073	0.118

* *p* < 0.05, ** *p* < 0.01. The dependent variable is “manager’s estimated causal contribution” as measured by the average score across each manager’s randomly assigned teams. Demographic factors include age, ethnicity, education and gender. PAGE and RMET scores are both standardized to have mean = 0 and *SD* = 1.

**Table 4:** Does emotion perception matter more at the start or end of the task?

	First Period	Second Period	Final Period
	(1)	(2)	(3)
PAGE	0.120	0.085	0.235 *
	(0.116)	(0.102)	(0.101)
Constant	−0.003	−0.013	0.043
	(0.113)	(0.100)	(0.099)
Observations	87	87	87
*R* ^2^	0.013	0.008	0.060

* *p* < 0.05. The dependent variable for each regression is the causal contribution of managers for a specific period of the game (*N* = 88). Column (1) represents the first third of the game; column (2) the middle period; and column (3) the last period. The dependent variable and the PAGE variable are standardized to have mean = 0 and *SD* = 1. The decrease in the sample of managers relative to Table 3 is a result of the data-collection process for Study 3. Group scores are captured at the end of the game and every time the manager changes task allocations. This typically happens in breaks (which occur after the first third and second third of the game). These intermediate scores allow for the estimation of causal contributions in each period. However, not every group makes a change during this period, which explains the decrease in manager numbers.

## Data Availability

The data and code for all studies, AI-generated images, and experiment materials are publicly available through an Open Science Foundation repository (https://osf.io/7a4xs/).

## Notes

1 Thanks to the advice of an anonymous reviewer, we acknowledge that it is currently unclear whether DALL-E can reliably generate culturally specific expressions. As such, the advantage of including ethnically diverse stimuli in the PAGE test is more likely to lie in promoting inclusivity for research participants rather than in capturing meaningful cultural display rules.

2 We conducted post hoc sensitivity analyses excluding participants with extremely low scores and those identified as likely inattentive responders based on response times. Across all thresholds, the reliability (Cronbach’s α) remained substantively unchanged. We thank an anonymous reviewer for this suggestion.

3 We thank an anonymous reviewer for suggesting this method. Parcels were constructed by averaging item difficulties, with mean difficulty levels carefully balanced across parcels (0.62–0.72).

4 We thank an anonymous reviewer for noting that that conducting exploratory and confirmatory analyses on the same sample does not constitute strict cross-validation and may capitalize on chance variance (Fokkema and Greiff 2017). Future work should replicate the factor structure in an independent sample.

5 This analysis accounts for the dependency structure of the data—whereby managers are present in multiple groups—by using a multilevel model with random effects for managers.

6 Specifically, repeated random assignment allows us to identify the average total contribution of each manager by taking the average of their group scores (as noted in Weidmann et al. 2024).

7 We thank an anonymous reviewer for pointing this out.

8 As above, we thank an anonymous reviewer for noting this.

9 We thank a second anonymous reviewer for this suggestion.

## Supplementary Materials

The following supporting information can be downloaded at https://www.mdpi.com/article/10.3390/jintelligence13090116/s1, Supporting text; Figure S1: Development pipeline of the PAGE test; Table S1: Confusion Matrix; Table S2: PAGE stimuli, target emotions, and distractors; Table S3: Item Difficulty Table; Table S4: Individual Item Characteristics; Table S5: Emotion Prompts for PAGE Stimulus Generation. Task materials for PAGE and RMET, and other surveys are also included in the supporting information.

## Appendix A

**Table A1:** Demographics of participants.

	Study 1	Study 2a	Study 2b ^	Study 3 *
**Number**	500	1010	741	116
**Ethnicity (%)**				
White	57.8	44.5	50.5	17.0
Black/African American	5.8	22.3	21.5	16.1
Latino/Hispanic ^0^	3.4	15.4	13.2	-
Asian °	20.0	17.9	14.8	56.2
Other/not reported	0.0	0.0	0.0	10.7
**Age**				
Mean (SD)	34.0 (9.4)	36.7 (9.1)	37.6 (9.1)	25.4 (4.5)
18–29 (%)	41.8	26.4	23.6	83.6
30–39 (%)	32.6	36.6	35.2	16.4
40–59 (%)	25.2	36.9	41.2	-
60–74 (%)	0.4	-	-	-
**Female (%)**	49	50	50	43
**Full-time workers (%)**	46	91	100	-
**Country**	US	US	US	UK

Notes: ^ Study 2b is a subset of study 2a. * This is the sample of managers in Study 3. The full experiment contained 555 participants. ° This includes ‘Asian British’; ^0^ in Study 3, which was done in the UK, this was not an option.

**Table A2:** PAGE image count by demographics.

Ethnicity	Count	Age	Count	Gender	Count
Caucasian	11	20–29	5	Female	17
Black	8	30–39	16	Male	18
Latino	9	40–59	13	Total	35
Asian	4	60	1		
Indian	2	Total	35		
Multi-racial	1				
Total	35				
