# Supplementary material for: Measuring Emotion Perception Ability Using AI-Generated Stimuli: Development and Validation of the PAGE Test
Source: J Intell. 2025 Sep 10;13(9):116. doi: 10.3390/jintelligence13090116 (PMC12471117; doi:10.3390/jintelligence13090116)
Supplement: Supplementary file 1 [file jintelligence-13-00116-s001.zip › jintelligence-3726029-supplementary.pdf]

Supporting Information for

Measuring emotion perception ability using AI-generated stimuli

This PDF file includes:

Supporting text

Figure S1

Tables S1 to S5

SI References

Other supporting materials for this manuscript include the following:

PAGE task (Study 2a, Study 2b)

RMET task (Study 2b)

Big 5 inventory (Study 3)

Demographics survey (Study 3)

Supporting text

Item-level CFA. To assess the unidimensionality of PAGE test, we also tested a one-factor confirmatory factor analysis (CFA) model. Model fit was evaluated by inspecting the comparative fit index (CFI), the Tucker–Lewis Index (TLI), and the root mean square error of approximation (RMSEA). The one-factor CFA model showed good fit ($\chi^2 = 884$, $df = 560$, $p < 0.001$, CFI = 0.829, TLI = 0.818, RMSEA = 0.024). Although the CFI and TLI are slightly lower than common acceptability threshold (0.9), the low RMSEA and satisfactory values of Cronbach's alpha overall suggest that the PAGE is a unidimensional test.

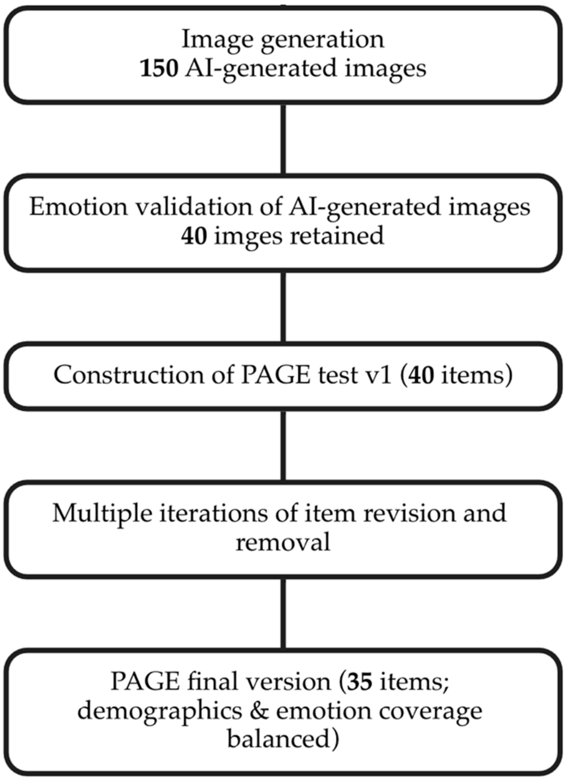

Figure S1 Development pipeline of the PAGE test.

Table S1 Confusion Matrix

Stimuli	Amu	Ang	Anx	Bor	Conc	Conf	Contemp	Contemp	Cont	Disa	Disg	Dou	Emb	Fea	Int	Joy	Pai	Pri	Sad	Sur	N
Amusement_35_Caucasian_Male	**77.3**	0.0	0.0	0.0	0.0	0.0	0.0	0.0	0.0	0.0	0.0	0.0	0.0	0.0	0.0	15.5	2.7	0.0	0.0	0.9	110
Anger_22_Caucasian_Female	0.0	**57.9**	0.0	0.0	0.7	0.0	0.0	10.7	0.0	10.7	17.1	0.0	0.0	0.0	0.0	0.0	0.7	0.0	0.7	0.0	140
Anger_30_Black_Male	0.0	**64.3**	0.7	0.0	5.0	0.0	0.0	12.9	0.7	5.0	8.6	0.0	0.0	0.7	0.0	0.0	0.7	0.0	0.0	0.7	140
Anger_40_Latino_Male	0.0	**66.7**	0.0	0.0	0.0	0.0	0.0	14.9	0.0	5.7	9.9	0.7	0.0	0.0	0.0	0.0	0.7	0.0	1.4	0.0	141
Anxiety_34_Caucasian_Male	0.0	0.0	**34.8**	0.7	3.5	9.2	10.6	0.0	1.4	6.4	0.0	1.4	3.5	9.2	0.7	0.0	2.1	0.0	7.8	0.7	141
Anxiety_40_Black_Male	0.0	0.0	**24.7**	0.0	1.3	10.0	2.0	1.3	0.0	5.3	4.0	14.0	1.3	13.3	0.7	0.0	1.3	0.0	6.0	0.7	150
Boredom_25_Caucasian_Female	0.0	0.0	0.9	**47.3**	18.2	7.3	2.7	0.0	0.0	0.9	0.9	9.1	0.0	0.0	1.8	0.0	6.4	1.8	2.7	0.0	110
Concentration_25_Caucasian_Female	0.0	7.1	0.7	6.4	**38.6**	1.4	9.3	17.9	4.3	2.1	0.0	4.3	0.0	0.7	2.9	0.0	0.0	0.7	0.0	0.0	140
Concentration_60_Multi-racial_Male	0.0	1.4	1.4	17.0	**38.3**	0.0	17.0	3.5	9.9	3.5	0.0	0.7	0.0	0.0	4.3	0.0	0.7	0.0	0.7	0.0	141
Confusion_40_Black_Female	0.9	0.9	0.9	0.0	0.0	**60.9**	0.0	2.7	0.0	1.8	6.4	17.3	0.0	0.0	0.0	0.0	0.0	0.0	0.0	5.5	110
Contemplation_30_Black_Male_1	0.0	0.0	1.4	10.7	14.3	2.1	**35.7**	1.4	4.3	0.7	0.0	14.3	0.0	0.7	10.0	0.0	0.0	0.0	0.0	0.0	140
Contemplation_30_Black_Male_2	0.0	1.4	0.7	10.0	17.1	3.6	**29.3**	5.0	0.7	13.6	0.7	12.1	0.0	0.0	5.0	0.0	0.7	0.0	0.0	0.0	140
Contempt_50_Black_Male	0.0	12.0	2.0	2.0	5.0	12.0	6.0	**26.0**	0.0	13.0	6.0	12.0	0.0	0.0	0.0	0.0	1.0	0.0	1.0	0.0	100
Contentment_35_Latino_Male	7.9	0.0	0.0	0.0	0.0	0.0	0.7	1.4	**40.0**	0.0	0.0	0.0	0.0	0.0	2.9	27.9	0.0	12.9	0.0	0.0	140
Contentment_45_Caucasian_Female	7.8	0.0	0.0	0.0	0.0	0.0	0.0	1.4	**46.1**	0.0	0.0	0.0	0.0	0.0	4.3	24.1	0.0	7.8	0.0	0.0	141
Contentment_55_Black_Male	6.4	0.0	0.0	0.0	0.0	0.0	0.0	3.6	**55.7**	0.0	0.0	0.0	0.0	0.0	1.4	17.1	0.0	7.1	0.0	0.0	140
Disappointment_30_Asian_Female	0.0	4.5	0.9	1.8	0.9	0.9	0.0	4.5	0.0	**40.0**	3.6	25.5	0.0	0.0	0.0	0.0	0.0	0.0	8.2	0.0	110
Disgust_35_Caucasian_Male	0.0	5.5	0.9	0.0	0.0	6.4	0.0	2.7	0.0	0.9	**77.3**	0.0	0.0	0.0	0.0	0.0	6.4	0.0	0.0	0.0	110
Disgust_45_Caucasian_Male	0.0	3.6	0.0	0.0	0.7	9.3	0.7	6.4	0.0	2.9	**76.4**	0.0	0.0	0.0	0.0	0.0	0.0	0.0	0.0	0.0	140
Doubt_30_Latino_Female_1	1.3	3.3	0.0	1.3	2.0	8.0	8.0	16.7	2.0	10.0	14.7	**25.3**	1.3	0.0	2.0	0.0	0.0	0.7	1.3	0.0	150
Doubt_30_Latino_Female_2	0.0	4.5	2.7	0.0	3.6	0.0	5.5	8.2	0.0	13.6	8.2	**43.6**	0.0	0.9	5.5	0.0	0.0	0.0	2.7	0.0	110
Embarrassment_20_Indian_Female	11.4	0.0	7.9	0.7	0.7	0.7	0.7	1.4	7.1	0.0	0.0	1.4	**38.6**	0.0	0.7	5.0	0.0	4.3	0.0	0.0	140
Fear_50_Asian_Female	0.0	0.0	15.5	0.0	0.0	7.3	0.0	0.0	0.0	1.8	1.8	0.9	0.0	**61.8**	0.0	0.0	0.0	0.0	0.0	8.2	110
Fear_50_Caucasian_Male	0.0	0.9	16.4	0.0	0.0	5.5	0.0	0.9	0.0	0.0	0.9	0.0	2.7	**50.9**	0.0	0.0	0.0	0.0	0.0	18.2	110
Interest_25_Latino_Male	3.6	0.0	0.0	0.0	3.6	0.0	10.9	0.9	9.1	0.0	0.0	1.8	0.0	0.0	**40.9**	0.0	0.0	14.5	0.0	0.0	110
Interest_50_Latino_Male	0.7	0.0	1.3	0.7	10.0	5.3	17.3	0.7	1.3	0.0	0.0	5.3	0.7	1.3	**28.0**	0.7	0.0	0.0	0.0	10.7	150
Joy_45_Latino_Female_1	22.1	0.0	0.0	0.0	0.0	0.0	0.0	0.0	0.0	0.0	0.0	0.0	0.0	0.0	0.7	**69.3**	0.0	0.0	0.0	0.7	140
Joy_45_Latino_Female_2	17.0	0.0	0.0	0.0	0.0	0.0	0.0	0.0	1.4	0.0	0.0	0.0	0.0	0.0	1.4	**58.2**	0.0	2.1	0.0	4.3	141
Pain_30_Black_Female	0.0	8.2	0.9	0.0	0.0	0.0	0.0	0.0	0.0	4.5	11.8	0.0	0.9	0.0	0.0	0.0	**50.9**	0.0	17.3	0.0	110
Pride_30_Asian_Male	5.5	0.0	0.0	0.0	0.0	0.0	0.9	3.6	24.5	0.0	0.0	0.0	0.0	0.0	5.5	2.7	0.0	**53.6**	0.0	0.0	110
Pride_30_Caucasian_Female	4.5	0.0	0.0	0.0	0.0	0.0	0.9	3.6	21.8	0.0	0.0	0.0	0.0	0.0	3.6	0.9	0.0	**63.6**	0.0	0.0	110
Sadness_30_Caucasian_Male	0.0	0.0	3.6	0.9	0.0	1.8	0.0	0.0	0.0	10.0	0.0	0.9	0.0	0.9	0.0	0.0	2.7	0.0	**69.1**	0.0	110
Sadness_30_Latino_Female	0.0	0.0	5.0	2.9	0.0	0.0	0.7	0.0	0.0	8.6	0.7	0.7	0.7	2.1	0.0	0.0	2.9	0.0	**65.0**	0.0	140
Surprise_30_Asian_Female	0.0	0.0	0.0	0.0	0.0	2.7	0.0	0.0	0.0	0.0	0.0	0.0	0.0	3.3	0.0	0.0	0.0	0.0	0.0	**86.7**	150
Surprise_47_Indian_Female	0.0	1.4	0.7	0.0	0.7	0.7	0.0	0.0	0.0	0.0	2.1	0.0	0.7	14.9	0.0	0.0	0.7	0.0	0.0	**64.5**	141

Note. Each cell shows the percentage of raters who selected a given emotion label for the corresponding stimulus (row); rows therefore sum to 100% apart from rounding. Cells shaded in green mark the emotion chosen most frequently, which in all cases is the intended (target) emotion for that stimulus. *N* is the number of raters who evaluated each image.

Table S2 PAGE stimuli, target emotions, and distractors

#	Stimuli	Target emotion	Distractors in multiple-choice test
1	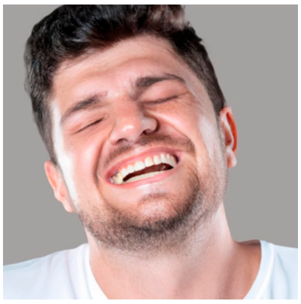	Amusement	Awe, Pleasure, Interest, Surprise, Relief
2	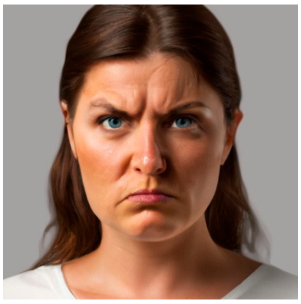	Anger	Pride, Pain, Disgust, Confusion, Shame
3	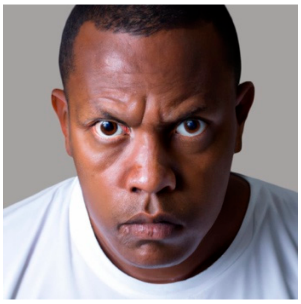	Anger	Pride, Pain, Confusion, Sadness, Disgust

4	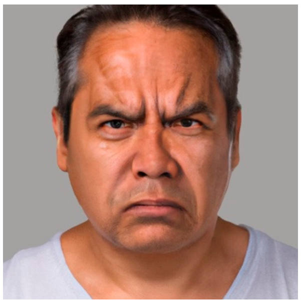	Anger	Amusement, Contempt, Sadness, Disappointment, Doubt
5	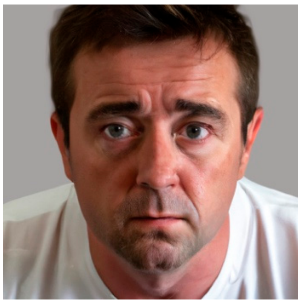	Anxiety	Contentment, Embarrassment, Contemplation, Confusion, Fear
6	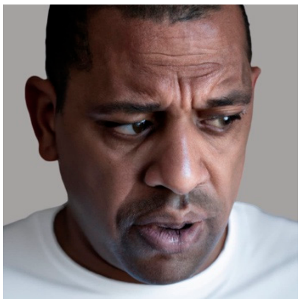	Anxiety	Contentment, Disappointment, Disgust, Relief, Boredom
7	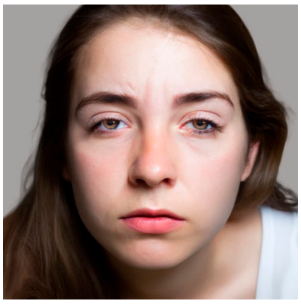	Boredom	Interest, Distress, Pleasure, Pain, Anger

8	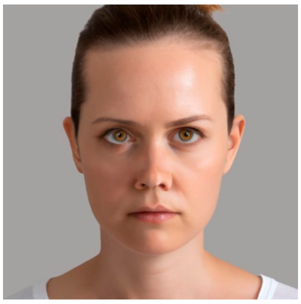	Concentration	Confusion, Doubt, Contentment, Disappointment, Interest
9	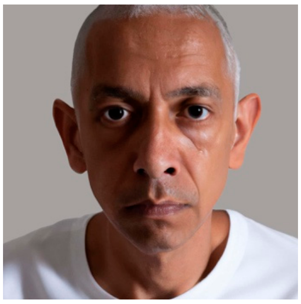	Concentration	Contentment, Interest, Contempt, Anger, Disappointment
10	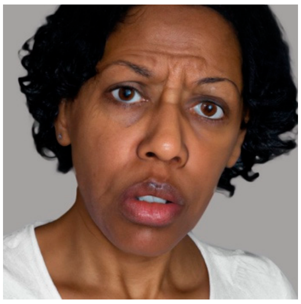	Confusion	Surprise, Interest, Anxiety, Doubt, Disgust
11	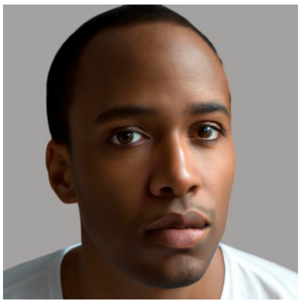	Contemplation	Confusion, Surprise, Disappointment, Interest, Contentment

12	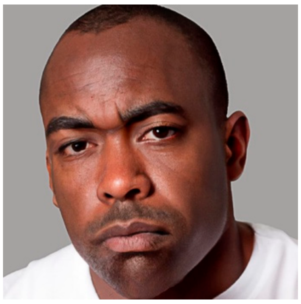	Contemplation	Anxiety, Relief, Surprise, Interest, Contentment
13	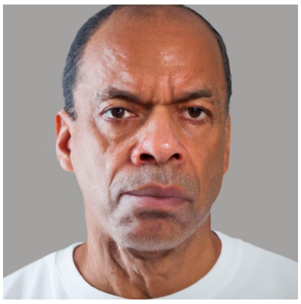	Contempt	Anxiety, Disgust, Confusion, Interest, Boredom
14	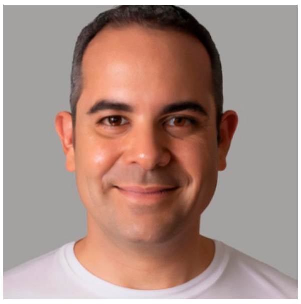	Contentment	Ecstasy, Pride, Desire, Contemplation, Contempt
15	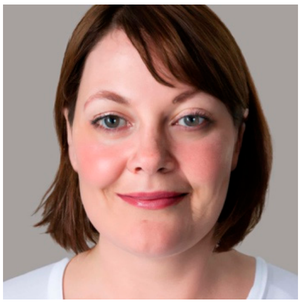	Contentment	Ecstasy, Disappointment, Pride, Contempt, Relief

16	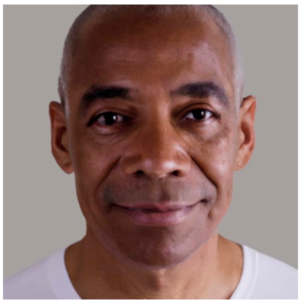	Contentment	Amusement, Pride, Desire, Joy, Contempt
17	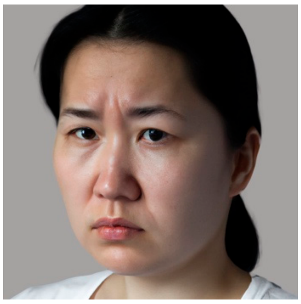	Disappointment	Confusion, Anger, Disgust, Contempt, Boredom
18	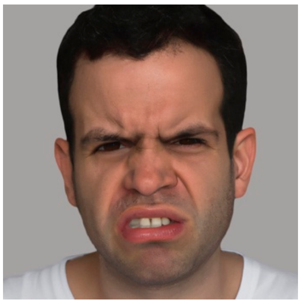	Disgust	Amusement, Pain, Contempt, Confusion, Anger
19	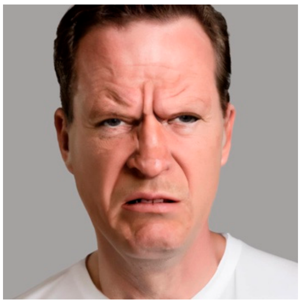	Disgust	Contemplation, Pain, Contempt, Confusion, Anger

20	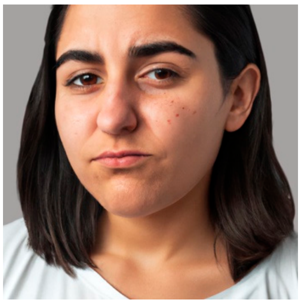	Doubt	Contentment, Confusion, Anger, Contemplation, Interest
21	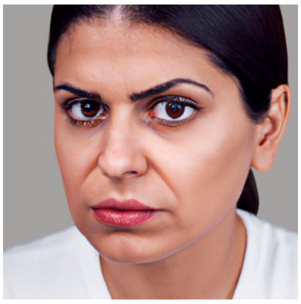	Doubt	Interest, Confusion, Boredom, Sadness, Anxiety
22	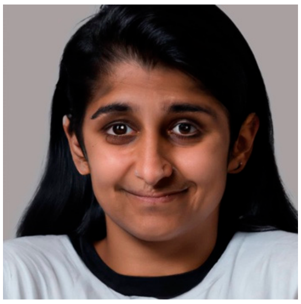	Embarrassment	Relief, Confusion, Pride, Anxiety, Shame
23	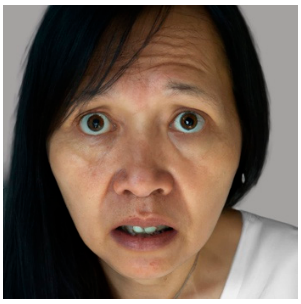	Fear	Awe, Confusion, Surprise, Anxiety, Shame

24	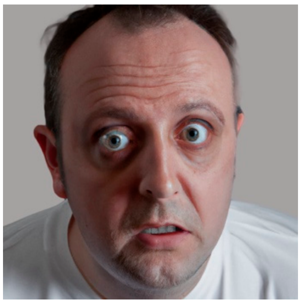	Fear	Anger, Confusion, Awe, Embarrassment, Pleasure
25	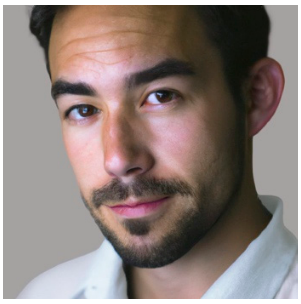	Interest	Amusement, Boredom, Doubt, Contemplation, Sympathy
26	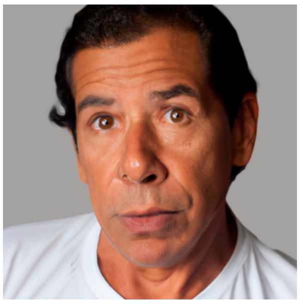	Interest	Doubt, Boredom, Embarrassment, Surprise, Disappointment
27	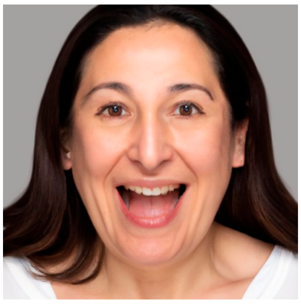	Joy	Awe, Surprise, Desire, Contentment, Confusion

28	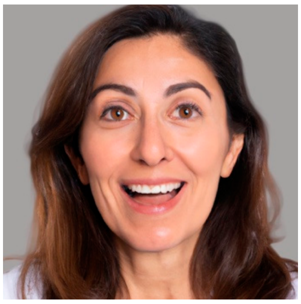	Joy	Surprise, Contentment, Desire, Confusion, Awe
29	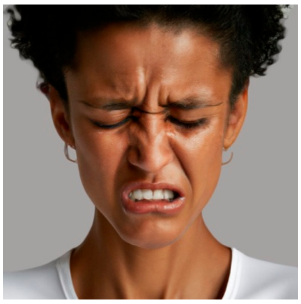	Pain	Pride, Disappointment, Anger, Embarrassment, Shame
30	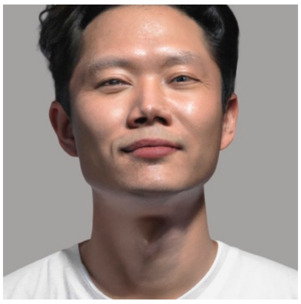	Pride	Amusement, Awe, Interest, Joy, Contempt
31	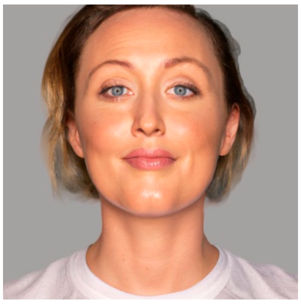	Pride	Amusement, Doubt, Interest, Contentment, Contempt

32	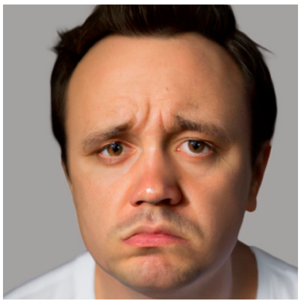	Sadness	Contentment, Anxiety, Confusion, Pain, Boredom
33	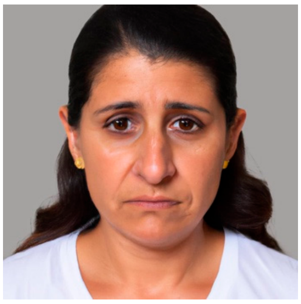	Sadness	Contentment, Anxiety, Disappointment, Pain, Boredom
34	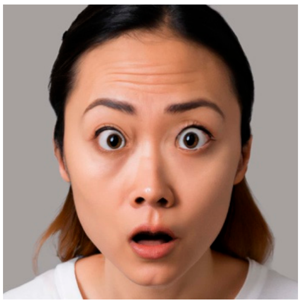	Surprise	Interest, Ecstasy, Confusion, Anger, Fear
35	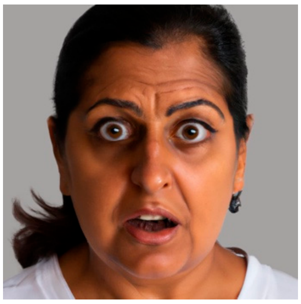	Surprise	Interest, Anger, Relief, Confusion, Disgust

Table S3 Item Difficulty Table

Item	Overall (n=1010)	Female	Male	White	Non_ White
Sadness_30_Caucasian_Male	87.2	87.4	87.1	88.9	85.9
Joy_45_Latino_Female_1	79.2	74.4	84.1	81.1	77.7
Confusion_40_Black_Female	67.2	73.4	61	71.7	63.6
Anger_30_Black_Male	84.2	83.6	84.7	86	82.7
Surprise_47_Indian_Female	89.1	89	89.3	90	88.4
Disgust_35_Caucasian_Male	74.1	76.7	71.4	76.6	72
Fear_50_Asian_Female	70	71.2	68.8	73.5	67.2
Boredom_25_ Caucasian _Female	64.9	59.2	70.6	61	67.9
Anxiety_40_Black_Male	64.6	63.1	66	66.8	62.7
Doubt_30_Latino_Female_1	65.7	65.1	66.4	63.3	67.7
Disappointment_30_Asian_Female	61.4	62.7	60	62.6	60.4
Interest_50_ Latino _Male	61.8	63.7	59.8	63.5	60.4
Embarrassment_20_Indian_Female	44.6	48.7	40.4	48.8	41.2
Pride_30_Asian_Male	66.6	65.7	67.6	73.3	61.3
Contentment_55_Black_Male	54.4	61.5	47.1	58.1	51.3
Fear_50_Caucasian_Male	71.1	71.4	70.8	75.7	67.4
Pride_30_Caucasian_Female	66.1	70.2	62	72.4	61.1
Contentment_35_ Latino _Male	64.8	70	59.4	69.9	60.6
Amusement_35_Caucasian_Male	61.8	63.7	59.8	64.1	59.9
Anxiety_34_Caucasian_Male	44.2	42.6	45.7	43.2	44.9
Contempt_50_Black_Male	47.7	49.9	45.5	51.2	44.9
Anger_22_Caucasian_Female	84.8	86	83.5	83.7	85.6
Contentment_45_Caucasian_Female	64.4	70.2	58.4	67.9	61.5
Sadness_30_Latino_Female	74.4	73.4	75.3	78.4	71.1
Doubt_30_Latino_Female _2	65.9	66.5	65.4	68.2	64.2
Surprise_30_Asian_Female	88.6	86.4	90.9	90.6	87
Interest_25_Latino _Male	59.3	59.8	58.8	61.7	57.4
Pain_30_Black_Female	61.9	63.5	60.2	62.4	61.5
Concentration_25_Caucasian_Female	70.7	70.6	70.8	71.5	70.1
Anger_40_Latino _Male	81.2	81.5	80.9	82.2	80.4
Joy_45_Latino _Female_2	72.7	68.6	76.7	73.7	71.8
Contemplation_30_Black_Male_1	50.8	53.6	47.9	50.1	51.3
Concentration_60_Multi-racial_Male	64.3	64.1	64.4	61.2	66.7
Disgust_45_Caucasian_Male	70.5	69.6	71.4	73.1	68.4
Contemplation_30_Black_Male_2	68.5	70.8	66.2	69.5	67.7

Note. The item labels are written in the format of 'emotion_age_ethnicity_gender' to indicate the emotion and demographic information of each face. For example, 'Sadness_30_Caucasian_Male' represents a face of 30 year old male expressing the emotion sadness.

Table S4 Individual Item Characteristics

Item	Loading_std	Correlation with total
Sadness_30_Caucasian_Male	0.63	0.37
Contentment_45_Caucasian_Female	0.59	0.37
Contentment_35_ Latino _Male	0.59	0.36
Pride_30_Asian_Male	0.56	0.37
Contentment_55_Black_Male	0.52	0.33
Surprise_47_Indian_Female	0.51	0.28
Pride_30_Caucasian_Female	0.48	0.32
Contempt_50_Black_Male	0.47	0.30
Fear_50_Caucasian_Male	0.42	0.27
Anger_30_Black_Male	0.41	0.24
Anger_22_Caucasian_Female	0.40	0.24
Interest_50_ Latino _Male	0.39	0.27
Disgust_35_Caucasian_Male	0.39	0.24
Fear_50_Asian_Female	0.37	0.24
Doubt_30_Latino_Female _1	0.37	0.25
Anxiety_40_Black_Male	0.37	0.24
Anxiety_34_Caucasian_Male	0.36	0.23
Confusion_40_Black_Female	0.35	0.23
Disappointment_30_Asian_Female	0.34	0.23
Doubt_30_Latino_Female _2	0.34	0.23
Boredom_25_ Caucasian _Female	0.32	0.23
Joy_45_Latino _Female_1	0.32	0.21
Embarrassment_20_Indian_Female	0.30	0.19
Concentration_60_Multi-racial_Male	0.30	0.20
*Amusement_35_Caucasian_Male	0.28	0.19
*Disgust_45_Caucasian_Male	0.27	0.18
*Contemplation_30_Black_Male_2	0.27	0.18
*Contemplation_30_Black_Male_1	0.26	0.19
*Interest_25_Latino _Male	0.26	0.18
*Surprise_30_Asian_Female	0.26	0.14
*Sadness_30_Latino_Female	0.26	0.17
*Concentration_25_Caucasian_Female	0.22	0.15
*Anger_40_Latino _Male	0.16	0.09
*Joy_45_Latino _Female_2	0.16	0.12
*Pain_30_Black_Female	0.11	0.08

Note: Items marked with * are those with both item–total correlations below 0.20 and standardized factor loadings below 0.30, indicating low discrimination. After removing these items, the internal consistency (Cronbach's α) was 0.72. The correlation with RMET remained similar: corrected correlation = 0.85; raw correlation = 0.65. We chose to retain these items to preserve representation of underrepresented racial/ethnic groups and to ensure coverage of rarely assessed emotions such as amusement, contemplation, and pain.

Table S5 Emotion Prompts for PAGE Stimulus Generation

**Emotion (Sources)**	**Prompt**	**Method**
Amusement (Keltner 1995)	"Generate a photorealistic image of a [age] [ethnicity] [gender] laughing with jaw dropping, head tilting backwards, with detailed skin texture and natural lighting, with highly realistic, well-proportioned eyes, with opened eyes. No body language, showing the face and shoulder, head oriented at the front, and looking at the camera. Plain grey background, wearing a white t-shirt."	facial actions
Anger (Ekman 2007)	"A realistic photo of a [age] [ethnicity] [gender] feeling very **angry**. Symmetric eyes. No body language, face in the middle, head oriented at the front, and staring at the camera. Plain grey background, wearing a white t-shirt"	emotion word
Anxiety (Perkins et al. 2012)	"Create a hyper-realistic image of a [age] [ethnicity] [gender] showing expression **anxiety**. Eyes looking sideways, frowned eyebrows, biting lips. Detailed skin texture and natural lighting. Wearing a white t-shirt. No body language, showing only the face, head oriented at the front, and staring at the camera. Plain grey background."	emotion word + facial actions
Boredom (Scherer and Ellgring 2007a; 2007b; Cordaro et al. 2020)	"Create a hyper-realistic image of a [age] [ethnicity] [gender] showing expression **boredom**. Eyelids dropping. Detailed skin texture and natural lighting. Wearing a white t-shirt. No body language, showing only the face, head oriented at the front, and staring at the camera. Plain grey background."	emotion word + facial actions
Concentration (Rozin and Cohen 2003)	" A realistic photo of a [age] [ethnicity] [gender] feeling very **concentrated**, clearly paying attention to something intently. No body language, face in the middle, head oriented at the front, and staring at the camera. Plain grey background, wearing a white t-shirt No body language, face in the middle, head oriented at the front, . Plain grey background, wearing a white t-shirt"	emotion word + emotion story
Confusion (Rozin and Cohen 2003)	"Create a hyper-realistic image of a [age] [ethnicity] [gender] showing a **confused** expression with slightly opened mouth. Detailed skin texture and natural lighting. No body language, showing only the face, head	emotion word + facial actions

	oriented at the front, and staring at the camera. Plain grey background, he is wearing a white t-shirt."	
Contemplation (Rozin and Cohen 2003)	"A realistic photo of a [age] [ethnicity] [gender] expressing the emotion **contemplation**, he is pondering life. No body language, face in the middle, head oriented at the front, and staring at the camera. Plain grey background, wearing a white t-shirt"	emotion word + emotion story
Contempt (Matsumoto and Ekman 2004)	"Create a hyper-realistic photo of a [age] [ethnicity] [gender] expressing **contempt**. Detailed skin texture and natural lighting. No body language, showing only the face, head oriented at the front, and staring at the camera. Plain grey background, wearing a white T-shirt."	emotion word
Contentment (Cordaro et al. 2016)	"A realistic photo of a [age] [ethnicity] [gender] experiencing a feeling of **well-being and delight**. His whole face and head in the middle. Plain grey background (leave some blank space around). He is wearing a white t-shirt. No body language, head oriented at the front, and staring at the camera."	Synonym of emotion word
Disappointment (Cordaro et al. 2016)	"Create a hyper-realistic image of a [age] [ethnicity] [gender] showing expression **disappointment**. Eyebrows slightly furrowed, lips pressed, eyes looking sideways. Detailed skin texture and natural lighting. Wearing a white t-shirt. No body language, showing only the face, head oriented at the front, and staring at the camera. Plain grey background."	emotion word + facial actions
Disgust (Ekman 2007)	"A realistic photo of a [age] [ethnicity] [gender] feeling **disgusted**. No body language, face in the middle, head oriented at the front, and staring at the camera. Plain grey background, wearing a white t-shirt"	emotion word
Doubt (Benitez-Quiroz, Wilbur, and Martinez 2016)	"Create a hyper-realistic image of a [age] [ethnicity] [gender] showing a **doubtful** expression with pressed lips. Detailed skin texture and natural lighting. No body language, showing only the face, head oriented at the front, and staring at the camera. Plain grey background, he is wearing a white t-shirt."	emotion word + facial actions
Embarrassment (Keltner 1995)	"A realistic photo of a [age] [ethnicity] [gender] caught **embarrassed** and blushing in a social gaffe. Her whole face and head in the middle. Plain grey background (leave some blank space around). She is wearing a white	emotion word + facial action + emotion story

	t-shirt. No body language, head oriented at the front, and staring at the camera."	
Fear (Ekman 2007)	"Create a hyper-realistic image of a [age] [ethnicity] [gender] showing expression **fear**. Wearing a white t-shirt. Detailed skin texture and natural lighting. No body language, showing only the face, head oriented at the front, and staring at the camera. Plain grey background,	emotion word
Interest (Reeve 1993)	"Create a hyper-realistic image of a [age] [ethnicity] [gender] showing expression **interest**. His eyebrows pulled straight up, eyes open wide, he has a small smile, his head tilts forward,. Detailed skin texture and natural lighting. No body language, showing only the face, head oriented at the front, and staring at the camera. Plain grey background, he is wearing a white t-shirt."	emotion word + facial action
Joy (Ekman 2007)	"A realistic photo of a [age] [ethnicity] [gender] expressing emotion **joy**, she is very happy at something unexpected. No body language, showing only the face, head oriented at the front, and staring at the camera. Plain grey background, wearing a white t-shirt""	emotion word + emotion story
Pain (Prkachin 1992)	"Create a hyper-realistic image of a [age] [ethnicity] [gender] showing a **painful** expression. Her eyes closed tightly, her lips tighten and pressed. Detailed skin texture and natural lighting. No body language, showing only the face, head oriented at the front, and staring at the camera. Plain grey background, he is wearing a white t-shirt.""	emotion word + facial action
Pride (Tracy and Robins 2004)	"Create a hyper-realistic image of a [age] [ethnicity] [gender] showing **pride**. His head holds high, jaw thrusts out, he has a small smile, lip pressed. Detailed skin texture and natural lighting. No body language, showing only the face, head oriented at the front, and staring at the camera. Plain grey background, wearing a white t-shirt."	emotion word + facial action
Sadness (Ekman 2007)	"A realistic photo of a [age] [ethnicity] [gender] showing a **sad** face when hearing an old friend's death. her whole face and head in the middle. Plain grey background (leave some blank space around). she is wearing a white t-shirt. No body language, face in the middle, head oriented at the front, and staring at the camera."	emotion word + emotion story

Surprise (Ekman 2007)	"A realistic photo of a [age] [ethnicity] [gender] showing a **surprised** face when hearing something she didn't expect. her whole face and head in the middle. Plain grey background (leave some blank space around). she is wearing a white t-shirt. No body language, face in the middle, head oriented at the front, and staring at the camera."	emotion word + emotion story

PAGE (short and long versions), RMET, and surveys

Construction of the short PAGE

To construct the short version of PAGE, we ranked items by their correlation with the total test score, selecting higher-ranking items while ensuring facial diversity. We balanced gender (8 male, 8 female), age (20-60 years), and ethnicity (six categories), and included both basic and complex emotions. The resulting 16-item version PAGE takes only 4 minutes to complete, offering a more practical option for time-constrained experiments. Items included in the short version are: 2, 5, 8, 9, 10, 12, 16, 18, 20, 23, 24, 26, 27, 29, 30, 34.

PAGE task instructions

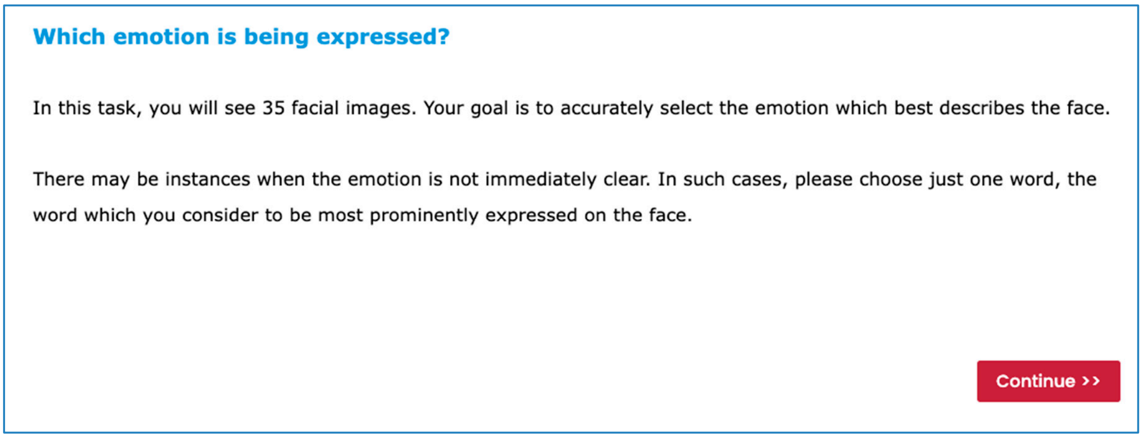

Under each image, 6 emotion words are presented. Before making your choice, make sure that you have read all 6 words. To know the definition of each emotion word, hover your cursor over **the specific word** for seconds and its definition will appear. See the example screenshot.

You can also look it up in the list of definitions. Please open this link in a new tab to reference during the survey. Note that not all emotions listed are expressed on these faces.

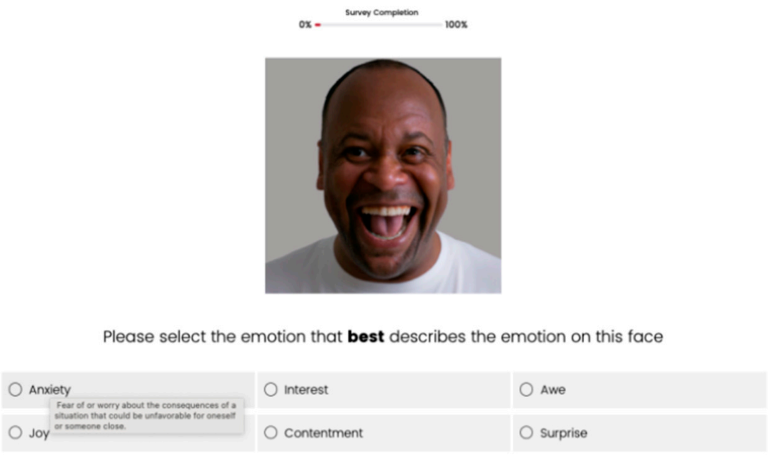

Continue >>

Most people take 6-8 minutes to complete the test.

Now you need to answer one simple practice questions to get familiar with the task format. Please take as much time as you need.

Continue >>

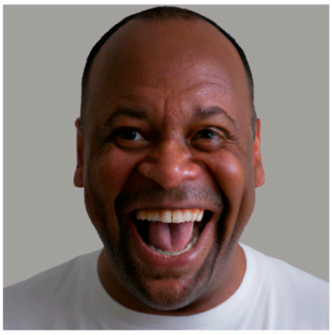

Please select the emotion that **best** describes the emotion on this face

- ○ Surprise
- ○ Contentment
- ○ Awe
- ○ Interest
- ○ Anxiety
- ○ Joy

Continue >>

PAGE items 1 – 35

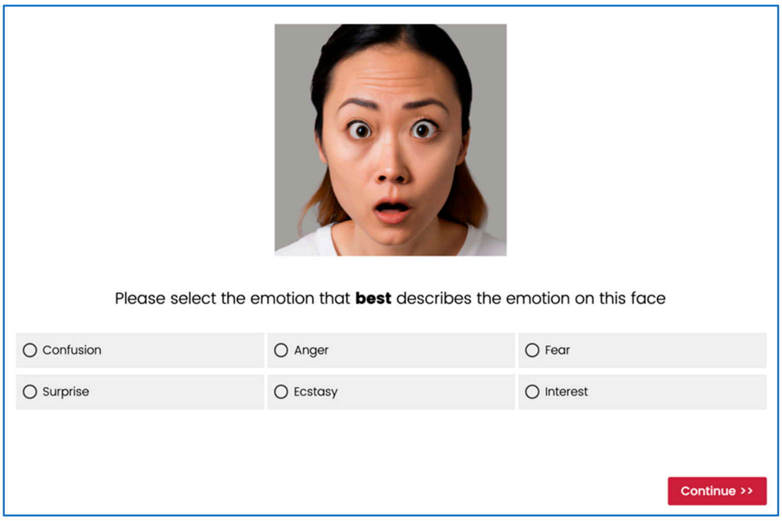

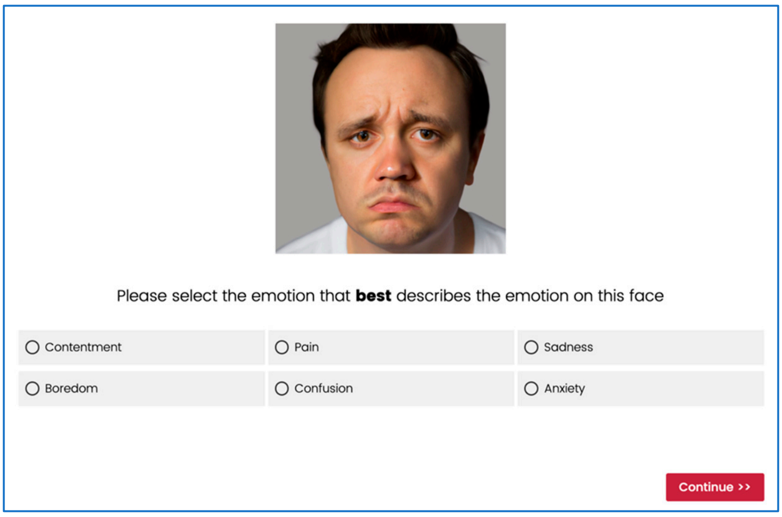

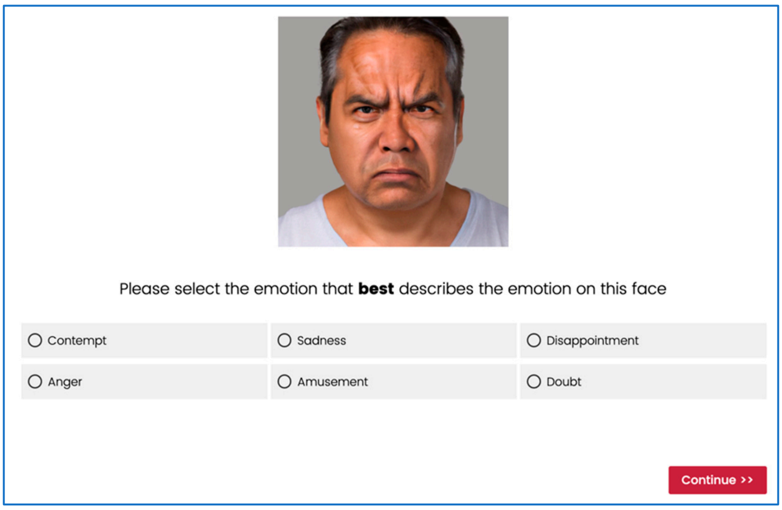

Please select the emotion that best describes the emotion on this face
Contempt
Sadness
Disappointment
Anger
Amusement
Doubt
Continue >>

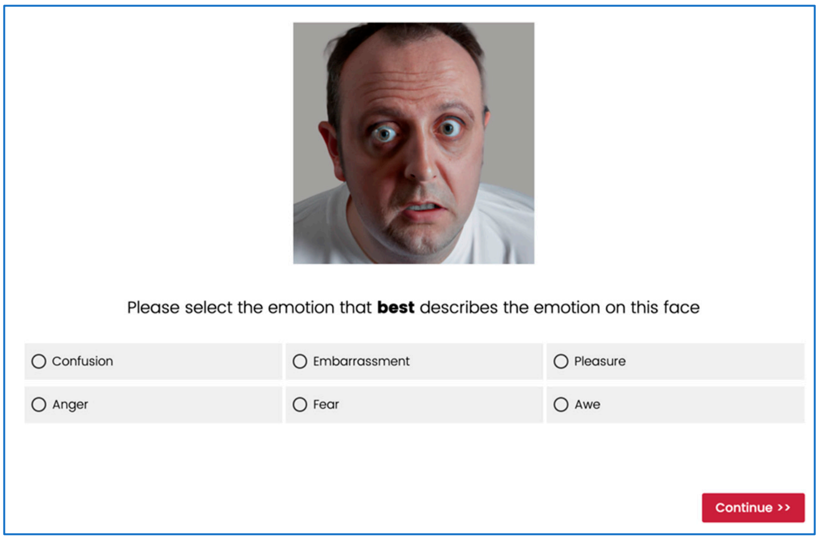

Please select the emotion that best describes the emotion on this face
Confusion
Embarrassment
Pleasure
Anger
Fear
Awe
Continue >>

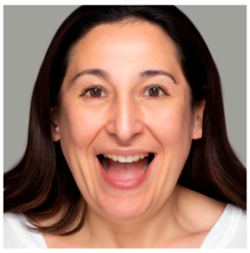

Please select the emotion that **best** describes the emotion on this face

○ Confusion	○ Awe	○ Desire
○ Joy	○ Contentment	○ Surprise

Continue >>

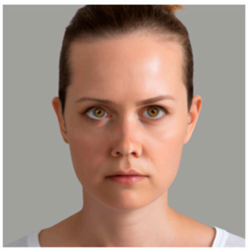

Please select the emotion that **best** describes the emotion on this face

○ Confusion	○ Interest	○ Disappointment
○ Concentration	○ Contentment	○ Doubt

Continue >>

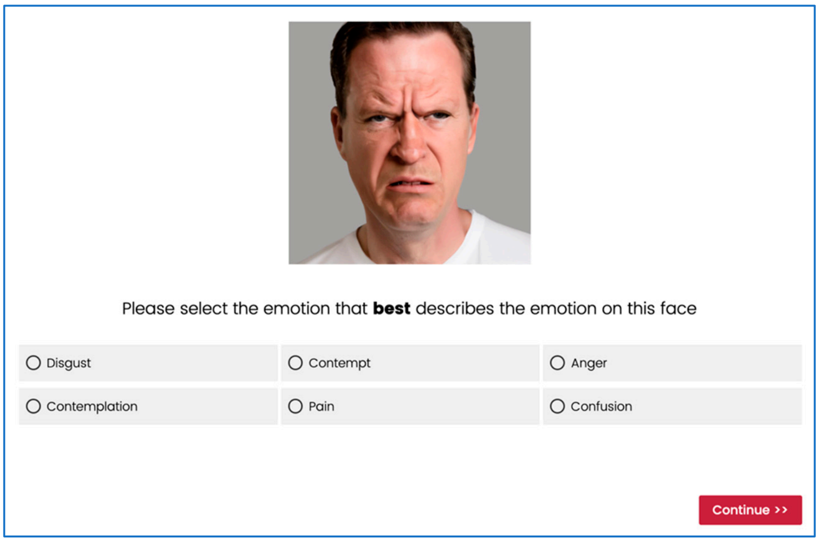
Please select the emotion that best describes the emotion on this face
Disgust
Contempt
Anger
Contemplation
Pain
Confusion
Continue >>

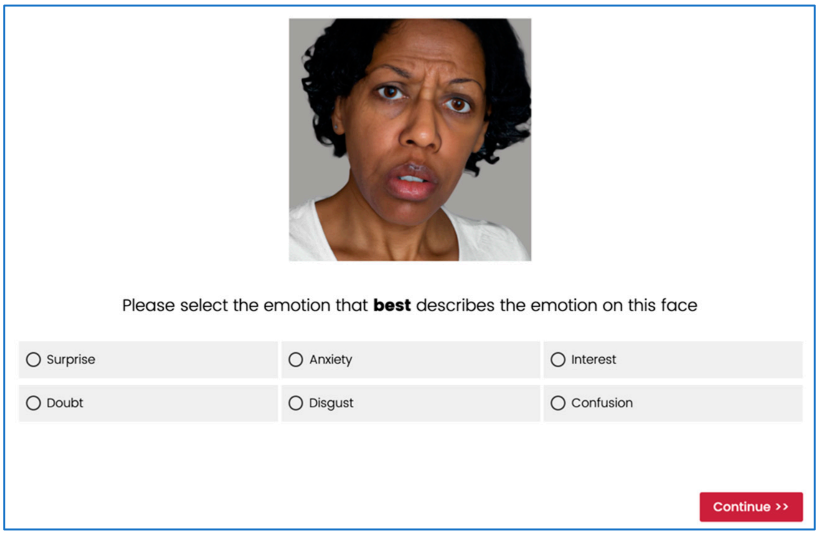
Please select the emotion that best describes the emotion on this face
Surprise
Anxiety
Interest
Doubt
Disgust
Confusion
Continue >>

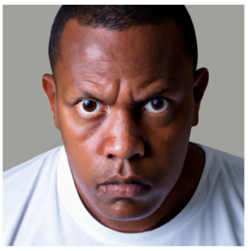

Please select the emotion that **best** describes the emotion on this face

○ Confusion	○ Anger	○ Sadness
○ Disgust	○ Pain	○ Pride

Continue >>

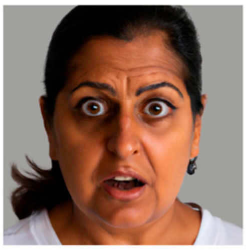

Please select the emotion that **best** describes the emotion on this face

○ Relief	○ Disgust	○ Confusion
○ Anger	○ Surprise	○ Interest

Continue >>

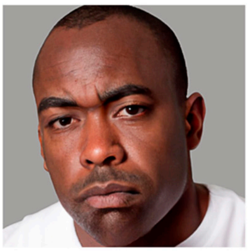

Please select the emotion that **best** describes the emotion on this face

○ Anxiety	○ Contentment	○ Interest
○ Surprise	○ Contemplation	○ Relief

Continue >>

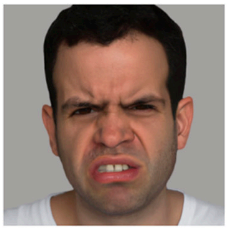

Please select the emotion that **best** describes the emotion on this face

○ Confusion	○ Pain	○ Anger
○ Disgust	○ Contempt	○ Amusement

Continue >>

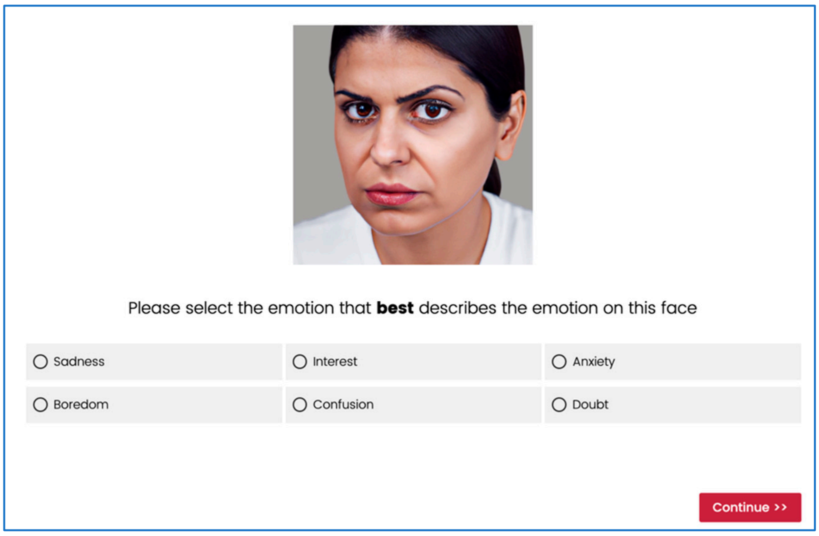
Please select the emotion that **best** describes the emotion on this face
Sadness
Interest
Anxiety
Boredom
Confusion
Doubt
Continue >>

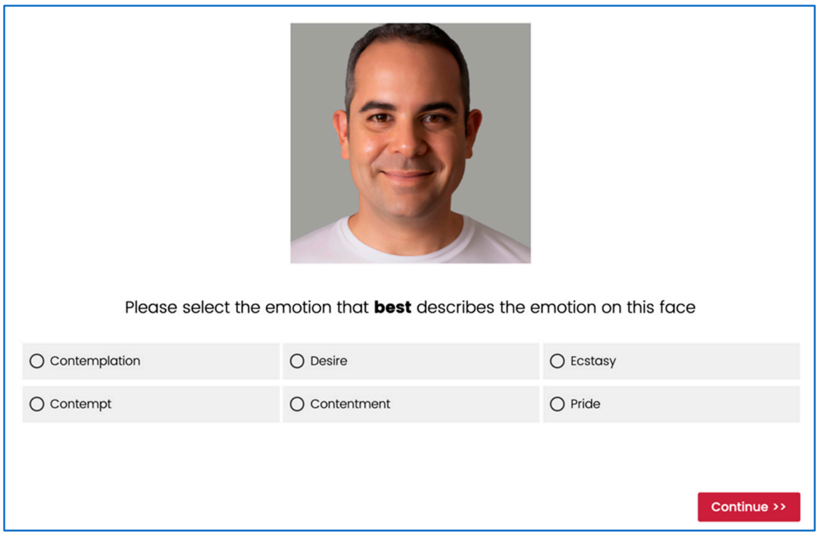
Please select the emotion that **best** describes the emotion on this face
Contemplation
Desire
Ecstasy
Contempt
Contentment
Pride
Continue >>

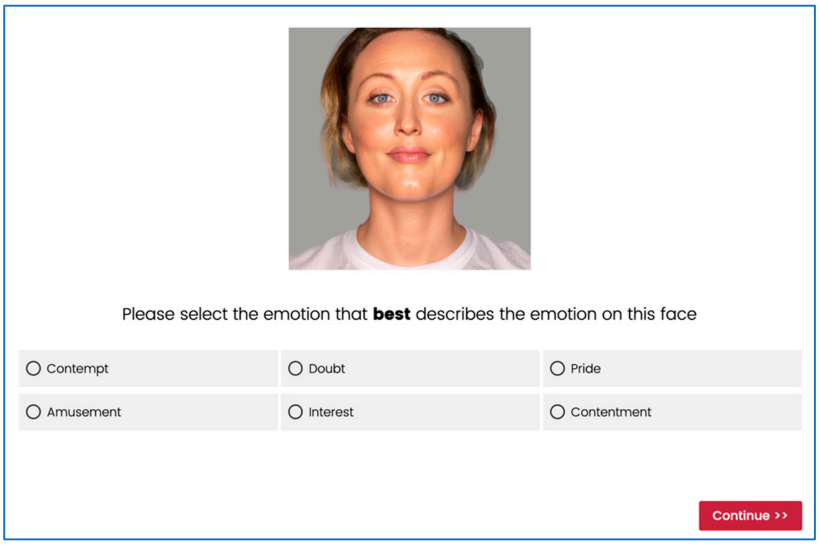
Please select the emotion that **best** describes the emotion on this face
Contempt
Doubt
Pride
Amusement
Interest
Contentment
Continue >>

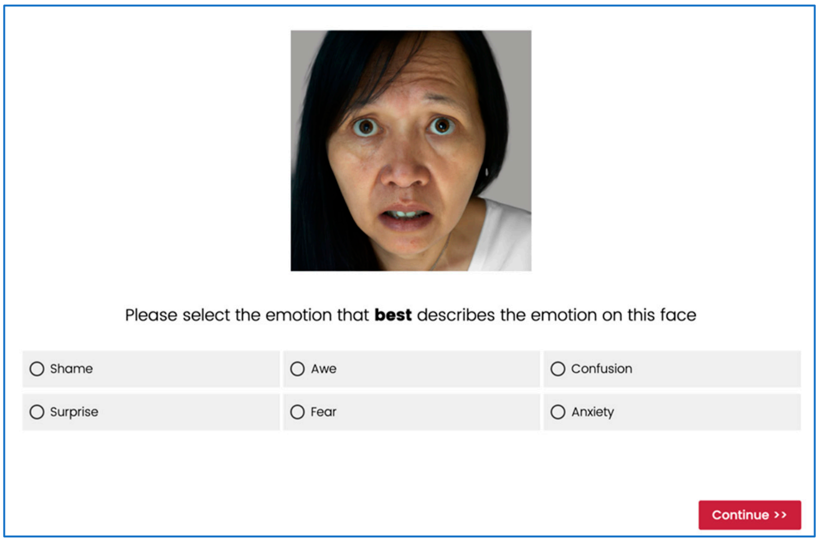
Please select the emotion that **best** describes the emotion on this face
Shame
Awe
Confusion
Surprise
Fear
Anxiety
Continue >>

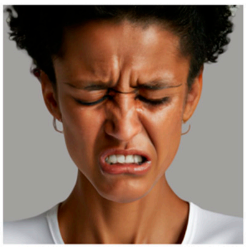

Please select the emotion that **best** describes the emotion on this face

Pain	Anger	Shame
Embarrassment	Pride	Disappointment

Continue >>

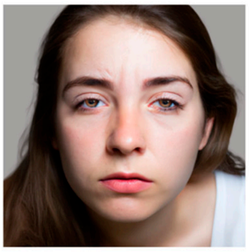

Please select the emotion that **best** describes the emotion on this face

Anger	Boredom	Interest
Distress	Pain	Pleasure

Continue >>

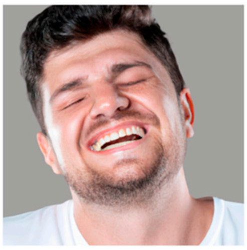

Please select the emotion that **best** describes the emotion on this face

○ Surprise	○ Awe	○ Interest
○ Amusement	○ Pleasure	○ Relief

Continue >>

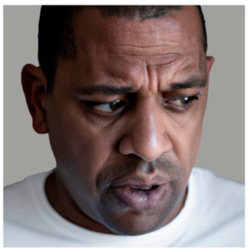

Please select the emotion that **best** describes the emotion on this face

○ Anxiety	○ Boredom	○ Disgust
○ Relief	○ Contentment	○ Disappointment

Continue >>

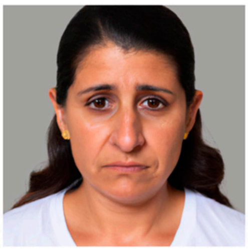

Please select the emotion that **best** describes the emotion on this face

○ Disappointment	○ Sadness	○ Pain
○ Boredom	○ Contentment	○ Anxiety

Continue >>

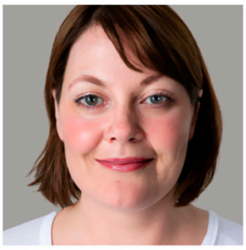

Please select the emotion that **best** describes the emotion on this face

○ Contentment	○ Ecstasy	○ Pride
○ Disappointment	○ Relief	○ Contempt

Continue >>

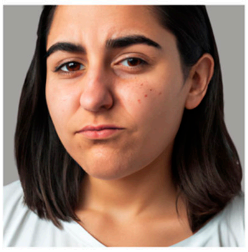

Please select the emotion that **best** describes the emotion on this face

○ Interest	○ Anger	○ Contemplation
○ Confusion	○ Doubt	○ Contentment

Continue >>

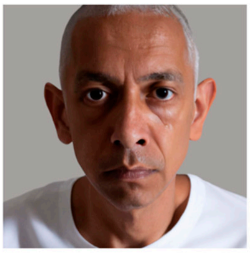

Please select the emotion that **best** describes the emotion on this face

○ Concentration	○ Contempt	○ Disappointment
○ Anger	○ Contentment	○ Interest

Continue >>

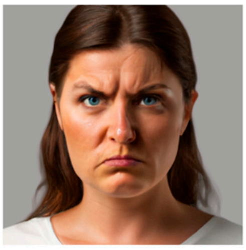

Please select the emotion that **best** describes the emotion on this face

◯ Shame	◯ Confusion	◯ Anger
◯ Disgust	◯ Pride	◯ Pain

Continue >>

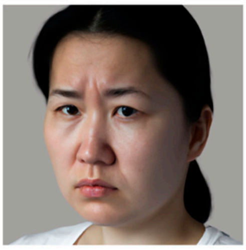

Please select the emotion that **best** describes the emotion on this face

◯ Boredom	◯ Contempt	◯ Disgust
◯ Anger	◯ Confusion	◯ Disappointment

Continue >>

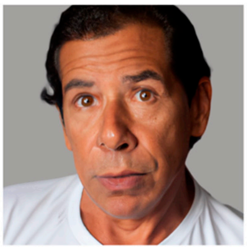

Please select the emotion that **best** describes the emotion on this face

○ Boredom	○ Surprise	○ Disappointment
○ Interest	○ Doubt	○ Embarrassment

Continue >>

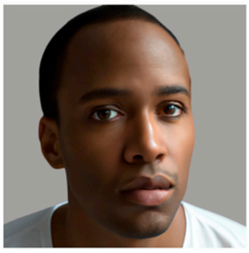

Please select the emotion that **best** describes the emotion on this face

○ Interest	○ Confusion	○ Contentment
○ Contemplation	○ Surprise	○ Disappointment

Continue >>

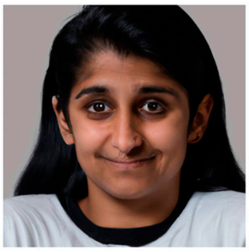

Please select the emotion that **best** describes the emotion on this face

○ Anxiety	○ Pride	○ Confusion
○ Embarrassment	○ Shame	○ Relief

Continue >>

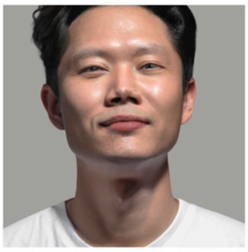

Please select the emotion that **best** describes the emotion on this face

○ Interest	○ Pride	○ Awe
○ Joy	○ Contempt	○ Amusement

Continue >>

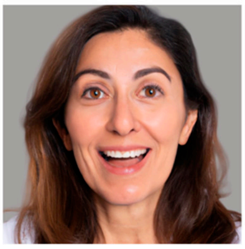

Please select the emotion that **best** describes the emotion on this face

○ Confusion	○ Joy	○ Contentment
○ Awe	○ Surprise	○ Desire

Continue >>

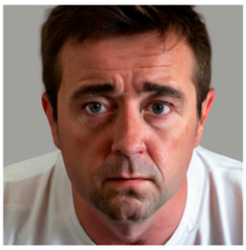

Please select the emotion that **best** describes the emotion on this face

○ Anxiety	○ Contentment	○ Contemplation
○ Embarrassment	○ Fear	○ Confusion

Continue >>

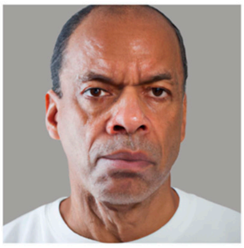

Please select the emotion that **best** describes the emotion on this face

◯ Boredom	◯ Disgust	◯ Confusion
◯ Contempt	◯ Interest	◯ Anxiety

Continue >>

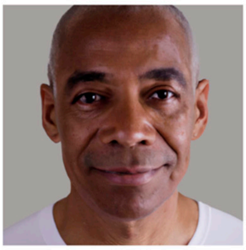

Please select the emotion that **best** describes the emotion on this face

◯ Contempt	◯ Contentment	◯ Amusement
◯ Desire	◯ Pride	◯ Joy

Continue >>

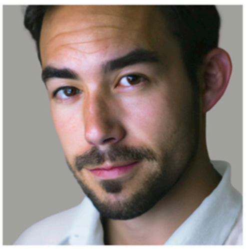

Please select the emotion that **best** describes the emotion on this face

◯ Boredom	◯ Doubt	◯ Amusement
◯ Sympathy	◯ Contemplation	◯ Interest

Continue >>

RMET Task instructions

Please re-enter your Prolific ID

Continue >>

Task 1: Read the Mind in the Eyes

On the following pages you will be presented with a series of 36 pictures of eyes. For each set of eyes, choose and select which word best describes what the person in the picture is **thinking or feeling**.

You may feel that more than one word is applicable but please choose just one word, the word which you consider to be most suitable.

Continue >>

Task 1: Read the Mind in the Eyes

On the following pages you will be presented with a series of 36 pictures of eyes. For each set of eyes, choose and select which word best describes what the person in the picture is **thinking or feeling**.

You may feel that more than one word is applicable but please choose just one word, the word which you consider to be most suitable.

Continue >>

Before making your choice, make sure that you have read all 4 words. To know the meaning of each emotion word, hover your cursor over **the specific word** for seconds and its definition, and one example sentence will appear. See the example screenshot.

You can also look it up in the list of definitions. Please open this link in a new tab to reference during the survey. Note that not all words listed are expressed by these eyes. Please try your best, thanks!

Please choose and select which word best describes what the person in the picture is **thinking or feeling.**

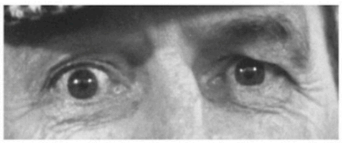

jealous	panicked	arrogant	hateful
○	○	○	○

envious. Tony was jealous of all the taller, better-looking boys in his class.

Continue >>

Practice

Please choose and select which word best describes what the person in the picture is **thinking or feeling.**

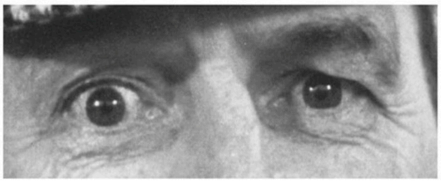

jealous	panicked	arrogant	hateful
○	○	○	○

Continue >>

RMET items 1 – 36

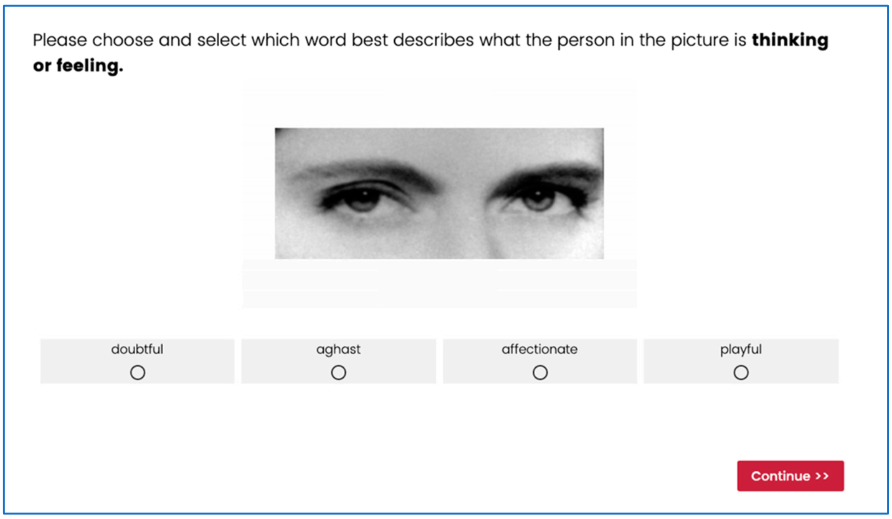

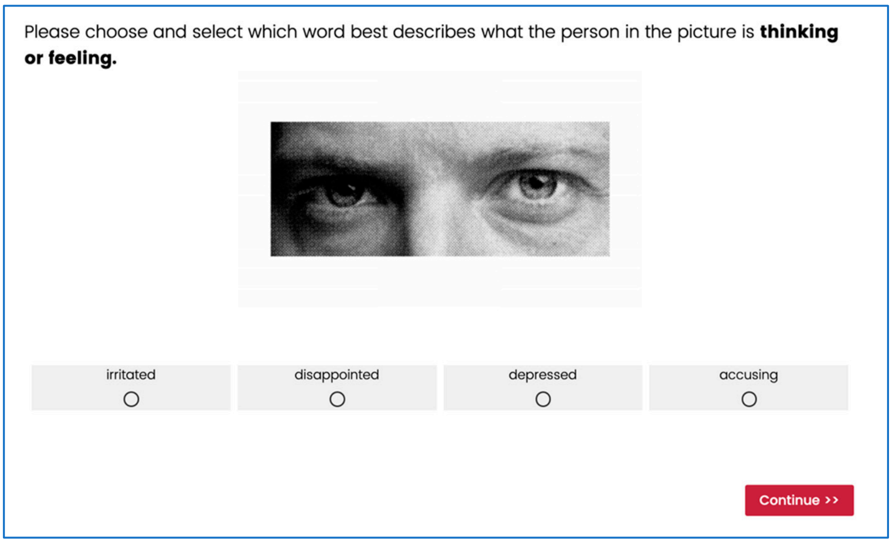

Please choose and select which word best describes what the person in the picture is **thinking or feeling.**

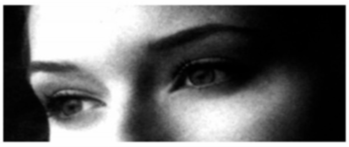

contemplative	flustered	encouraging	amused
○	○	○	○

Continue >>

Please choose and select which word best describes what the person in the picture is **thinking or feeling.**

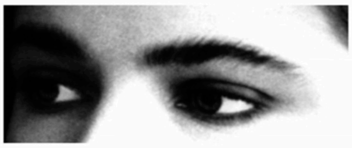

preoccupied	annoyed	hostile	horrified
○	○	○	○

Continue >>

Please choose and select which word best describes what the person in the picture is **thinking or feeling.**

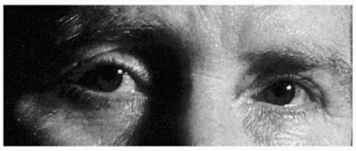

pensive	hostile	irritated	excited
○	○	○	○

Continue >>

Please choose and select which word best describes what the person in the picture is **thinking or feeling.**

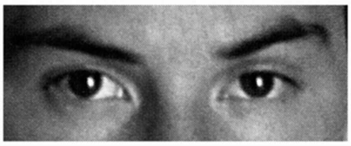

apologetic	defiant	contented	curious
○	○	○	○

Continue >>

Please choose and select which word best describes what the person in the picture is **thinking or feeling.**

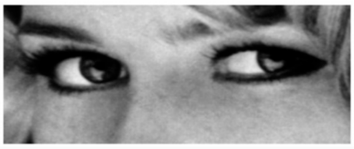

terrified	baffled	distrustful	aghast
○	○	○	○

Continue >>

Please choose and select which word best describes what the person in the picture is **thinking or feeling.**

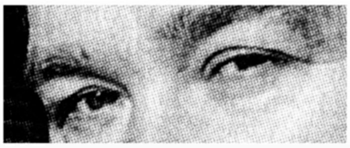

embarrassed	skeptical	indifferent	dispirited
○	○	○	○

Continue >>

Please choose and select which word best describes what the person in the picture is **thinking or feeling.**

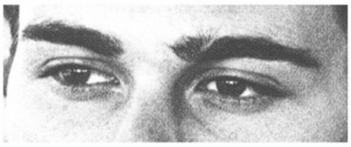

sympathetic	thoughtful	encouraging	irritated
○	○	○	○

Continue >>

Please choose and select which word best describes what the person in the picture is **thinking or feeling.**

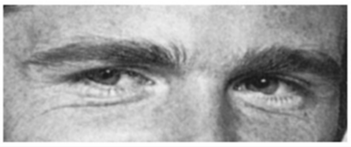

aghast	bored	cautious	insisting
○	○	○	○

Continue >>

Please choose and select which word best describes what the person in the picture is **thinking or feeling.**

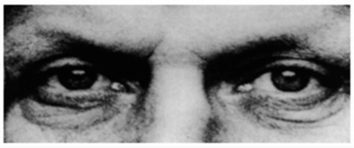

alarmed	bewildered	serious	ashamed
○	○	○	○

Continue >>

Please choose and select which word best describes what the person in the picture is **thinking or feeling.**

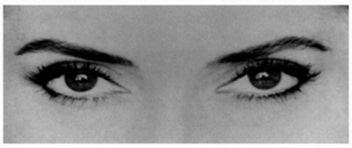

aghast	decisive	amused	bored
○	○	○	○

Continue >>

Please choose and select which word best describes what the person in the picture is **thinking or feeling.**

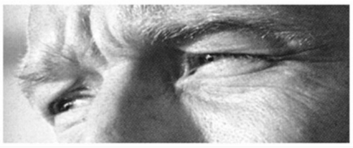

despondent ○ relieved ○ excited ○ shy ○

Continue >>

Please choose and select which word best describes what the person in the picture is **thinking or feeling.**

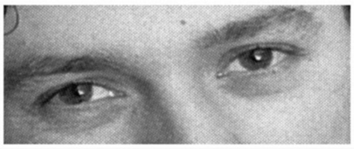

dominant ○ friendly ○ guilty ○ horrified ○

Continue >>

Please choose and select which word best describes what the person in the picture is **thinking or feeling.**

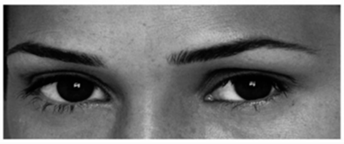

grateful	arrogant	sarcastic	tentative
○	○	○	○

Continue >>

Please choose and select which word best describes what the person in the picture is **thinking or feeling.**

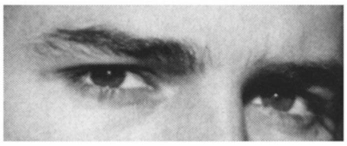

embarrassed	fantasizing	concerned	guilty
○	○	○	○

Continue >>

Please choose and select which word best describes what the person in the picture is **thinking or feeling.**

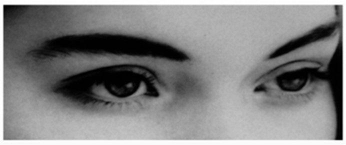

aghast	reflective	irritated	impatient
○	○	○	○

Continue >>

Please choose and select which word best describes what the person in the picture is **thinking or feeling.**

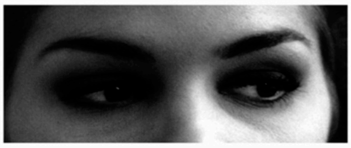

grateful	insisting	imploring	preoccupied
○	○	○	○

Continue >>

Please choose and select which word best describes what the person in the picture is **thinking or feeling.**

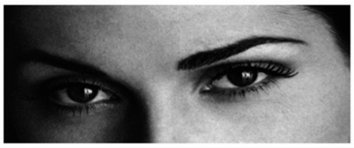

dispirited	ashamed	joking	confident
○	○	○	○

Continue >>

Please choose and select which word best describes what the person in the picture is **thinking or feeling.**

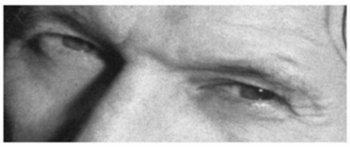

indecisive	nervous	ashamed	suspicious
○	○	○	○

Continue >>

Please choose and select which word best describes what the person in the picture is **thinking or feeling.**

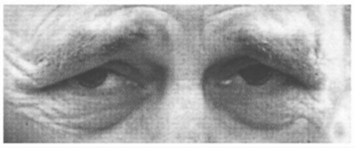

annoyed	arrogant	terrified	upset
○	○	○	○

Continue >>

Please choose and select which word best describes what the person in the picture is **thinking or feeling.**

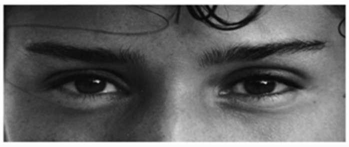

bored	comforting	irritated	playful
○	○	○	○

Continue >>

Please choose and select which word best describes what the person in the picture is **thinking or feeling.**

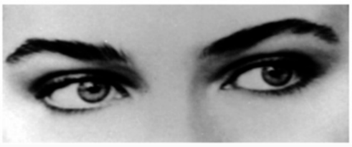

reassuring	cautious	joking	arrogant
○	○	○	○

Continue >>

Please choose and select which word best describes what the person in the picture is **thinking or feeling.**

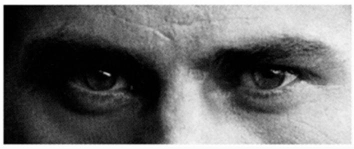

relaxed	amused	insisting	joking
○	○	○	○

Continue >>

Please choose and select which word best describes what the person in the picture is **thinking or feeling.**

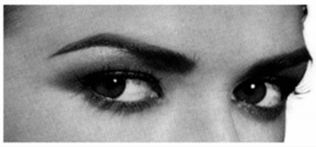

contented	joking	affectionate	interested
○	○	○	○

Continue >>

Please choose and select which word best describes what the person in the picture is **thinking or feeling.**

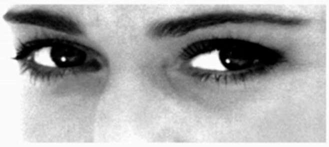

incredulous	panicked	interested	despondent
○	○	○	○

Continue >>

Please choose and select which word best describes what the person in the picture is **thinking or feeling.**

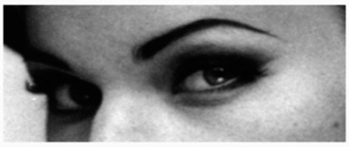

hostile	grateful	flirtatious	disappointed
○	○	○	○

Continue >>

Please choose and select which word best describes what the person in the picture is **thinking or feeling.**

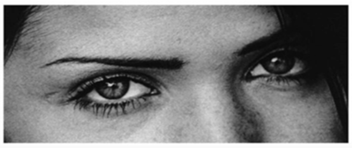

convinced	joking	desire	flustered
○	○	○	○

Continue >>

Please choose and select which word best describes what the person in the picture is **thinking or feeling.**

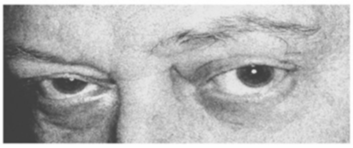

alarmed	shy	hostile	anxious
○	○	○	○

Continue >>

Please choose and select which word best describes what the person in the picture is **thinking or feeling.**

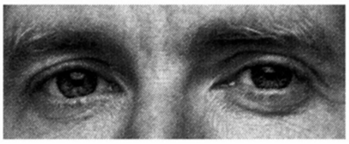

terrified	flirtatious	regretful	amused
○	○	○	○

Continue >>

Please choose and select which word best describes what the person in the picture is **thinking or feeling.**

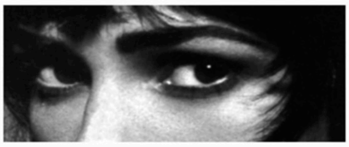

nervous	puzzled	contemplative	insisting
○	○	○	○

Continue >>

Please choose and select which word best describes what the person in the picture is **thinking or feeling.**

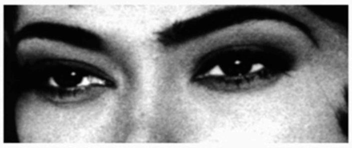

aghast	impatient	alarmed	fantasizing
○	○	○	○

Continue >>

Please choose and select which word best describes what the person in the picture is **thinking or feeling.**

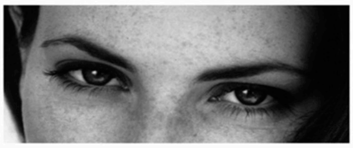

confused	panicked	embarrassed	fantasizing
○	○	○	○

Continue >>

Please choose and select which word best describes what the person in the picture is **thinking or feeling.**

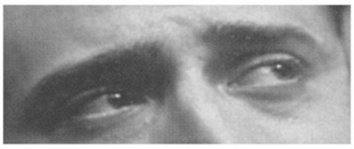

friendly	sarcastic	worried	irritated
○	○	○	○

Continue >>

Please choose and select which word best describes what the person in the picture is **thinking or feeling.**

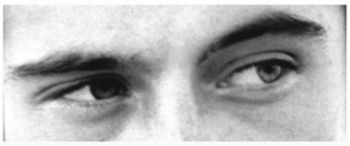

apologetic	uneasy	dispirited	friendly
○	○	○	○

Continue >>

Please choose and select which word best describes what the person in the picture is **thinking or feeling.**

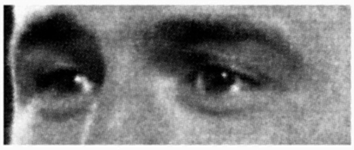

anticipating	shy	threatening	decisive
○	○	○	○

Continue >>

Big 5 inventory in Study 3 (Gosling, Rentfrow, and Swann 2003)

Here are a number of personality traits that may or may not apply to you.

Please write a number next to each statement to indicate the extent to which *you agree or disagree with that statement*. You should rate the extent to which the pair of traits applies to you, even if one characteristic applies more strongly than the other.

1 – Disagree strongly

2 – Disagree moderately

3 – Disagree a little

4 – Neither agree or disagree

5 – Agree a little

6 – Agree moderately

7 – Agree strongly

I *see myself as:*

1. Extraverted, enthusiastic.

2. Critical, quarrelsome.

3. Dependable, self-disciplined.

4. Anxious, easily upset.

5. Open to new experiences, complex.

6. Reserved, quiet.

7. Sympathetic, warm.

8. Disorganized, careless.

9. Calm, emotionally stable.

10. Conventional, uncreative.

Demographic questionnaire for Study 3

Note: for a full description of Study 3 materials, please see (Weidmann et al. 2024)

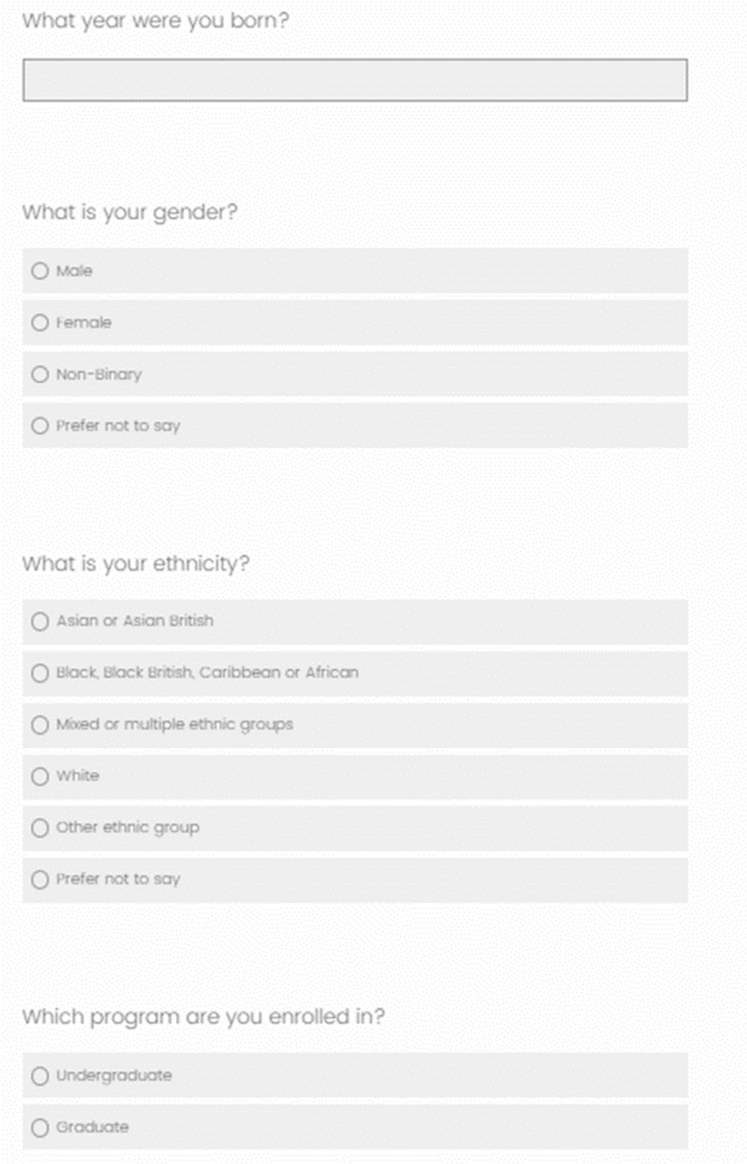

What year were you born?

What is your gender?

- Male
- Female
- Non-Binary
- Prefer not to say

What is your ethnicity?

- Asian or Asian British
- Black, Black British, Caribbean or African
- Mixed or multiple ethnic groups
- White
- Other ethnic group
- Prefer not to say

Which program are you enrolled in?

- Undergraduate
- Graduate

SI References

Benitez-Quiroz, C. Fabian, Ronnie B. Wilbur, and Aleix M. Martinez. 2016. "The Not Face: A Grammaticalization of Facial Expressions of Emotion." *Cognition* 150 (May):77–84. https://doi.org/10.1016/j.cognition.2016.02.004.

Cordaro, Daniel T., Marc Brackett, Lauren Glass, and Craig L. Anderson. 2016. "Contentment: Perceived Completeness across Cultures and Traditions." *Review of General Psychology* 20 (3): 221–35. https://doi.org/10.1037/gpr0000082.

Cordaro, Daniel T., Rui Sun, Shanmukh Kamble, Niranjan Hodder, Maria Monroy, Alan Cowen, Yang Bai, and Dacher Keltner. 2020. "The Recognition of 18 Facial-Bodily Expressions across Nine Cultures." *Emotion* 20 (7): 1292–1300. https://doi.org/10.1037/emo0000576.

Ekman, Paul. 2007. "The Directed Facial Action Task:Emotional Responses Without Appraisal." In *Handbook of Emotion Elicitation and Assessment*, edited by James A Coan and John J B Allen, 47–53. Oxford University PressNew York, NY. https://doi.org/10.1093/oso/9780195169157.003.0004.

Gosling, Samuel D, Peter J Rentfrow, and William B Swann. 2003. "A Very Brief Measure of the Big-Five Personality Domains." *Journal of Research in Personality* 37 (6): 504–28. https://doi.org/10.1016/S0092-6566(03)00046-1.

Keltner, Dacher. 1995. "Signs of Appeasement: Evidence for the Distinct Displays of Embarrassment, Amusement, and Shame." *Journal of Personality and Social Psychology* 68 (3): 441–54. https://doi.org/10.1037/0022-3514.68.3.441.

Matsumoto, David, and Paul Ekman. 2004. "The Relationship Among Expressions, Labels, and Descriptions of Contempt." *Journal of Personality and Social Psychology* 87 (4): 529–40. https://doi.org/10.1037/0022-3514.87.4.529.

Perkins, Adam M., Sophie L. Inchley-Mort, Alan D. Pickering, Philip J. Corr, and Adrian P. Burgess. 2012. "A Facial Expression for Anxiety." *Journal of Personality and Social Psychology* 102 (5): 910–24. https://doi.org/10.1037/a0026825.

Prkachin, Kenneth M. 1992. "The Consistency of Facial Expressions of Pain: A Comparison across Modalities." *Pain* 51 (3): 297–306. https://doi.org/10.1016/0304-3959(92)90213-U.

Reeve, Johnmarshall. 1993. "The Face of Interest." *Motivation and Emotion* 17 (4): 353–75. https://doi.org/10.1007/BF00992325.

Rozin, Paul, and Adam B. Cohen. 2003. "High Frequency of Facial Expressions Corresponding to Confusion, Concentration, and Worry in an Analysis of Naturally Occurring Facial Expressions of Americans." *Emotion* 3 (1): 68–75. https://doi.org/10.1037/1528-3542.3.1.68.

Scherer, Klaus R., and Heiner Ellgring. 2007a. "Are Facial Expressions of Emotion Produced by Categorical Affect Programs or Dynamically Driven by Appraisal?" *Emotion* 7 (1): 113–30. https://doi.org/10.1037/1528-3542.7.1.113.

———. 2007b. "Multimodal Expression of Emotion: Affect Programs or Componential Appraisal Patterns?" *Emotion* 7 (1): 158–71. https://doi.org/10.1037/1528-3542.7.1.158.

Tracy, Jessica L., and Richard W. Robins. 2004. "Show Your Pride: Evidence for a Discrete Emotion Expression." *Psychological Science* 15 (3): 194–97. https://doi.org/10.1111/j.0956-7976.2004.01503008.x.

Weidmann, Ben, Joseph Vecci, Farah Said, David J. Deming, and Sonia R. Bhalotra. 2024. "How Do You Find a Good Manager?" Working Paper. Working Paper Series. National Bureau of Economic Research. https://doi.org/10.3386/w32699.
